# The European cancer burden in 2020: Incidence and mortality estimates for 40 countries and 25 major cancers

**DOI:** 10.1016/j.ejca.2021.07.039

**Published:** 2021-11

**Authors:** Tadeusz Dyba, Giorgia Randi, Freddie Bray, Carmen Martos, Francesco Giusti, Nicholas Nicholson, Anna Gavin, Manuela Flego, Luciana Neamtiu, Nadya Dimitrova, Raquel Negrão Carvalho, Jacques Ferlay, Manola Bettio

**Affiliations:** a European Commission, Joint Research Centre (JRC), Ispra, Italy; b Cancer Surveillance Branch, International Agency for Research on Cancer (IARC), Lyon, France; c Northern Ireland Cancer Registry, Belfast, United Kingdom

**Keywords:** Cancer registries, Cancer, Europe, Incidence, Mortality, Estimation

## Abstract

**Introduction:**

Europe is an important focus for compiling accurate and up-to-date world cancer statistics owing to its large share of the world's total cancer burden. This article presents incidence and mortality estimates for 25 major cancers across 40 individual countries within European areas and the European Union (EU-27) for the year 2020.

**Methods:**

The estimated national incidence and mortality rates are based on statistical methodology previously applied and verified using the most recently collected incidence data from 151 population-based cancer registries, mortality data and 2020 population estimates.

**Results:**

Estimates reveal 4 million new cases of cancer (excluding non-melanoma skin cancer) and 1.9 million cancer-related deaths. The most common cancers are: breast in women (530,000 cases), colorectum (520,000), lung (480,000) and prostate (470,000). These four cancers account for half the overall cancer burden in Europe. The most common causes of cancer deaths are: lung (380,000), colorectal (250,000), breast (140,000) and pancreatic (130,000) cancers. In EU-27, the estimated new cancer cases are approximately 1.4 million in males and 1.2 million in females, with over 710,000 estimated cancer deaths in males and 560,000 in females.

**Conclusion:**

The 2020 estimates provide a basis for establishing priorities in cancer-control measures across Europe. The long-established role of cancer registries in cancer surveillance and the evaluation of cancer control measures remain fundamental in formulating and adapting national cancer plans and pan-European health policies. Given the estimates are built on recorded data prior to the onset of coronavirus disease 2019 (COVID-19), they do not take into account the impact of the pandemic.

## Highlights

• The 2020 incidence estimates derived from 151 population-based cancer registries' data.
• Over 4 million new cancer cases and 1.9 million cancer-related deaths are estimated in Europe for 2020.
• Half the overall cancer diagnoses are from breast , colorectal , lung and prostate cancers.
• The most common cancer-related deaths are from lung , colorectal , breast and pancreatic cancers.
• The 2020 estimates are key to assess and monitor cancer-control measures across Europe.

## 1 Introduction

Cancer accounts for a greater number of deaths among persons aged under 65 years than any other disease in the member states of the European Union (EU) [1]. Cancer is a major public concern on the continent: almost one-quarter of all global cancer cases occur in Europe, which is home to only one-tenth of the world's population [2,3]*.* In economic terms, cancer cost the EU almost €97 billion in 2018 [4]. Estimates of cancer burden at the national level are based on population-based cancer registry data and provide an important motivator for cancer-control policies. In Europe, the individual members of the European Network of Cancer Registries (ENCR, http://www.encr.eu/) are the institutions hosting the national or regional cancer-registry information systems providing this baseline information.

European estimates of the cancer burden have been made available for over 30 years [[5], [6], [7], [8], [9], [10], [11], [12]]. The latest figures for 2020 are presented herein, and they are based on the collaboration between the European Commission's Joint Research Centre (JRC, which also serves as the ENCR secretariat), the Cancer Surveillance Branch of the International Agency for Research on Cancer (IARC, which also serves as the Secretariat of the International Association of Cancer Registries – IACR) with the support of the ENCR and IACR.

Estimated incidence and mortality figures are based on several data sources, namely: the historical time series available in the European Cancer information System (ECIS) of the European Commission [13] and the data submitted to the project ‘Cancer Incidence in Five Continents Vol. XI (CI5-XI)’ [14] – for the incidence predictions; the World Health Organisation (WHO) database [15] – for the mortality predictions; ECIS, Eurostat [16] and the United Nation (UN) Population Division sources [3] – for the population data.

The 2020 cancer-burden estimates are described for the 25 most common cancer sites for each of the 39 UN-defined European countries [3] and Cyprus. In addition, aggregated results are provided for the EU-27 Member States; the four UN-defined European areas (Central and Eastern, Northern, Southern and Western Europe) [3]; and the whole of Europe.

The complete set of estimates for the 25 cancers, with additional cancer entities, is available from the ECIS web application (https://ecis.jrc.ec.europa.eu/) and from IARC's Global Cancer Observatory (GCO, https://gco.iarc.fr/).

## 2 Materials and methods

### 2.1 Data sources

To predict the estimated values of cancer burden, we considered regional and national cancer incidence, mortality, and population data broken down by sex and 18 age groups (0–4, 5–9, …, 80–84, 85 years and over).

For the national incidence predictions, subnational and national incidence data from 2003 to 2018 were provided by 151 European cancer registries (20 national and 131 regional registries) participating in two studies: (a) IARC, CI5-XI [14], and (b) ENCR-JRC Cancer Incidence and Mortality in Europe [https://encr.eu/encr-jrc-project] (from which the ECIS data are compiled). Regional mortality data were also provided by most of the cancer registries for the equivalent years of incidence data. For the national mortality predictions, the national mortality data from 2004 to 2018 were retrieved from the World Health Organisation (WHO) database [15]

Other incidence and mortality data were collected for selected countries. Aggregated incidence data for the Nordic European countries were taken from the NORDCAN database of the Association of Nordic Cancer Registries [17]; for Luxembourg, data were taken from a published report [18]; for the Russian Federation, the incidence data (up to 2018) were extracted from the series of reports published by the Moscow Research Oncological Institute [19]; other incidence and mortality data were also considered from a published report of the Montenegro cancer registry [20].

The population data were provided by cancer registries, or retrieved from Eurostat [16] and the United Nations (UN) [3]. In addition, projected populations for the year 2020 were taken from either Eurostat or UN data. In the following subsections of Materials and Methods, we provide the key definitions and methods used in compiling the European cancer estimates; further details are provided in Ferlay *et al.*, 2019 [2].

#### 2.1.1 Cancer site definition (incidence and mortality data)

Estimated cancer incidence and mortality results are presented for 25 selected cancer entities defined according to the 10th edition of the International Classification of Diseases (ICD-10, version 2010) [21], namely: lip, oral cavity and pharynx (ICD-10 C00–14); oesophagus (C15); stomach (C16); colorectum (including anus, (C18–21); liver (C22); gallbladder (C23); pancreas (C25); larynx (C32); lung (including trachea, C33–34); melanoma of skin (C43); breast (C50; females only); cervix uteri (C53); corpus uteri (C54); ovary (C56); prostate (C61); testis (C62); kidney (including renal pelvis, C64–65); bladder (C67); brain and other central nervous system (CNS) (C70–72): thyroid (C73); Hodgkin lymphoma (C81); non-Hodgkin lymphoma (NHL) (C82–86, C96); multiple myeloma (including immunoproliferative diseases, C88+C90); leukaemia (C91–95); and all cancers combined, excluding non-melanoma skin cancer (C00–97, except C44). This definition of cancer entities is the same as that used in the previous estimates and in the current worldwide estimates included in IARC's Global Cancer Observatory.

Selected ICD-10 unspecified cancer groupings [2] were redistributed to specific categories by year, sex and age following procedures already used for 2018 estimates [12]. The "unspecified cancers" group (ICD-10 categories C76–80 + C97) were not reallocated to specific categories but kept in a separated cancer entity.

The category of ‘all cancers combined’, excluding non-melanoma skin cancer, was then obtained by summing the estimated counts of new cancer cases or deaths for each individual cancer site in a given country, by sex and age together with the corresponding estimates of the residual category "other and unspecified cancers".

#### 2.1.2 Cancer site specifications in mortality data

To maximise comparability across European countries, certain corrections had to be applied in some instances to the mortality data. Owing to the incompleteness of death registration in a few European countries for the period under study, we needed to adjust the associated WHO mortality data with corresponding estimated completeness as reported by WHO (see Table 1). A further mortality data correction was performed for all deaths coded as ill-defined categories (ICD-10 "R" category, chapter XVIII) by redistributing them *pro rata* across cancer causes (ICD-10 "C" category, malignant neoplasms) and all other causes (excluding injuries), by year, sex, and age.

**Table 1:** Completeness of national mortality data (percent), coverage of incidence data (percent) and methods of estimation.

Country	Mortality					Incidence			
	Coverage (%)	Ill-defined (%)^a^	Method^b^		Exceptions^c^	Coverage (%)	Method^d^		Exceptions^c^
Albania	55	11/19	1	2000–2009 ↗ 2020	Kaposi sarcoma; anus, liver, vulva, vagina, and penile cancers from Croatia and Serbia	0	3B	Estimated from mortality with M:I ratios from cancer registries in Southern Europe model^e^	
Austria	100	4/3	1	2008–2017 ↗ 2020	Cervical and corpus uteri cancers estimated using survival (source EUROCARE-5)	100	1	2003–2012 ↗ 2020	Non-melanoma skin cancers from Tyrol and Vorarlberg (2010–2012 = 2020)
Belarus	100	4/13	2A	2018 = 2020		100	1	2003–2012 ↗ 2020	Prostate cancer (2010–2012 = 2020)
Belgium	100	6/8	1	2007–2016 ↗ 2020	Cervical and corpus uteri cancers estimated using survival (source EUROCARE-5)	100	1	2004–2013 ↗ 2020	
Bosnia and Herzegovina	93	7/7	2A	2011, 2014, 2016 = 2020		40	3A	Estimated from mortality (all ages)^g^ with M:I ratios from Republika of Srpska cancer registry (2008–2012)	
Bulgaria	100	3/4	1	2006–2015 ↗ 2020	Mesothelioma estimated using survival (source EUROCARE-5)	100	1	2004–2013 ↗ 2020	
Croatia	100	1/1	1	2008–2017 ↗ 2020	Cervical and corpus uteri cancers estimated using survival (source EUROCARE-5)	100	1	2003–2012 ↗ 2020	Non-melanoma skin cancers from Bosnia and Herzegovina, Republika of Srpska and Slovenia cancer registries (2008–2012 = 2020)
Cyprus^h^	74	3/4	1	2007–2016 ↗ 2020		70	1	2003–2012 ↗ 2020	Thyroid cancer (2010–2012 = 2020)
Czechia	100	2/1	1	2008–2017 ↗ 2020		100	1	2004–2013 ↗ 2020	
Denmark	100	8/10	1	2006–2015 ↗ 2020	Cervical and corpus uteri cancers estimated using survival (source NORDCAN)	100	1	2007–2016 ↗ 2020	Anus, vulva, vagina, non-melanoma skin and penile cancers, mesothelioma, Kaposi sarcoma (not available in NORDCAN) and brain and central nervous system tumours from Danish cancer registry
Estonia	100	2/2	1	2009–2018 ↗ 2020		100	1	2003–2012 ↗ 2020	Prostate cancer (2010–2012 = 2020)
Finland	100	1/1	1	2008–2017 ↗ 2020		100	1	2007–2016 ↗ 2020	Anus, vulva, vagina, non-melanoma skin and penile cancers, mesothelioma, Kaposi sarcoma (not available in NORDCAN) and brain and central nervous system tumours from Finnish cancer registry
France	100	10/12	1	2007–2016 ↗ 2020	Cervical and corpus uteri cancers estimated using survival (source EUROCARE-5)	18	3A	Estimated from mortality with M:I ratios from 15 French cancer registries	Thyroid cancer from 8 French cancer registries (2003–2012 ↗ 2020) Kaposi sarcoma, breast, prostate and childhood cancers from 15 French cancer registries (2008–2012 = 2020) Non-melanoma skin cancers from Haut-Rhin cancer registry (2003–2012 ↗ 2020)
Germany	100	4/3	1	2008–2017 ↗ 2020	Cervical and corpus uteri cancers estimated using survival (source EUROCARE-5)	73	1	2003–2012 ↗ 2020 (incidence from 8 German cancer registries)	Kaposi sarcoma and childhood cancers (2008–2012 = 2020)
Greece	100	5/8	1	2007–2016 ↗ 2020	Kaposi sarcoma; mesothelioma; vulva, vagina and penile cancers (2014–2016 = 2020)	0	3B	Estimated from mortality with M:I ratios from cancer registries in Southern Europe model^e^	Kaposi sarcoma, non-melanoma and childhood cancers (2018–2012 = 2020)
Hungary	100	0/0	1	2008–2017 ↗ 2020		0	3B	Estimated from mortality with M:I ratios from national cancer registries in Eastern Europe model^i^	Thyroid cancer and Hodgkin lymphoma (2008–2012 = 2020) Childhood cancers from paediatric cancer registry (2008–2012 = 2020)
Iceland	100	3/1	1	2009–2018 ↗ 2020		100	1	2007–2016 ↗ 2020	Anus, vulva, vagina, non-melanoma skin and penile cancers, mesothelioma, Kaposi sarcoma (not available in NORDCAN) and brain and central nervous system tumours from Iceland cancer registry
Ireland	100	0/0	1	2007–2015 ↗ 2020		100	1	2003–2012 ↗ 2020	Prostate cancer (2010–2012 = 2020)
Italy	100	2/3	1	2007–2016 ↗ 2020	Cervical and corpus uteri cancers estimated using survival (source EUROCARE-5)	50	3A	Estimated from mortality with M:I ratios from 33 Italian cancer registries	Breast and thyroid cancers from 13 Italian cancer registries (2003–2012 ↗ 2020) Kaposi sarcoma, prostate and childhood cancers from 38 cancer registries (2008–2012 = 2020) Non-melanoma skin cancers from 5 Italian cancer registries (2003–2012 ↗ 2020)
Latvia	100	1/1	1	2006–2015 ↗ 2020		100	1	2003–2012 ↗ 2020	
Lithuania	100	3/1	1	2009–2018 ↗ 2020		100	1	2003–2012 ↗ 2020	Prostate cancer (2010–2012 = 2020)
Luxembourg	100	3/4	1	2007–2016 ↗ 2020		0	3B	Estimated from mortality (2012–2016)^j^ with M:I ratios from cancer registries in Belgium and France	Average number of cases (2007–2016) lower than recorded cases of national pathology-based register^k^ (2013) completed by recorded incidence cases (source national pathology-based register)
Malta	100	1/1	1	2007–2016 ↗ 2020		100	1	2004–2013 ↗ 2020	
Moldova	83	1/0	1	2009–2018 ↗ 2020		0	3B	Estimated from mortality with M:I ratios from Ukrainian (2013–2015) cancer registry	
Montenegro^l^	94	18/17	2A	2013 = 2020		0	2A	2013 = 2020	
Netherlands	100	4/5	1	2008–2017 ↗ 2020		100	1	2004–2013 ↗ 2020	Liver and pancreatic cancer cases (2004–2013) completed by cancer deaths (source WHO)
North Macedonia	100	7/7	1	2006–2013 ↗ 2020		0	3B	Estimated from mortality with M:I ratios from cancer registries in Southern Europe model^e^	
Norway	100	6/6	1	2007–2016 ↗ 2020	Cervical and corpus uteri cancers estimated using survival (source NORDCAN)	100	1	2007–2016 ↗ 2020	Anus, vulva, vagina, non-melanoma skin and penile cancers, mesothelioma, Kaposi sarcoma (not available in NORDCAN) and brain and central nervous system tumours from Norwegian Cancer Registry
Poland	100	12/10	1	2008–2017 ↗ 2020	Cervical and corpus uteri cancers estimated using survival (source EUROCARE-5)	33	3A	Estimated from mortality with M:I ratios from 6 Polish cancer registries	Breast, prostate and thyroid cancers from the 6 Polish cancer registries (2003–2012 ↗ 2020) Kaposi sarcoma, non-melanoma skin and childhood cancers from the 6 Polish cancer registries (2008–2012 = 2020)
Portugal	100	6/8	1	2008–2017 ↗ 2020	Cervical and corpus uteri cancers estimated using survival (source EUROCARE-5)	99	3A	Estimated from mortality with M:I ratios from 4 Portuguese local cancer registries (2008–2011)	Liver, pancreatic and lung cancers cases completed by cancer deaths (by registry, year, sex and age) Kaposi sarcoma, non-melanoma skin, breast, prostate, thyroid and childhood cancers from the 4 Portuguese cancer registries (2008–2011 = 2020)
Romania	100	1/1	1	2008–2017 ↗ 2020		22	3B	Estimated from mortality with M:I ratios from cancer registries in Eastern Europe model^i^	Childhood cancers from Cluj and Timisoara cancer registries (2008–2011 = 2020)
Russian Federation	100	7/12	1	2009–2018 ↗ 2020	Lip, oral cavity and pharynx, colon, rectum, and anus cancers from St-Petersburg and Arkhangelsk Russian cancer registries	100	1	2009-2018^m^ ↗ 2020	Anus and non-melanoma skin cancers, mesothelioma and Kaposi sarcoma from St-Petersburg and Arkhangelsk Russian cancer registries (2008–2012 = 2020) Oesophageal, gastric, liver, pancreatic and lung cancer cases (2009–2018) completed by cancer deaths (source WHO)
Serbia^n^	95	6/5	1	2008-2017^o^ ↗ 2020		57	3B	Estimated from mortality with M:I ratios from cancer registries in Eastern Europe model^i^	Kaposi sarcoma and childhood cancers estimated from Central Serbia cancer registry (2009–2011 = 2020)
Slovakia	100	2/2	1	2005–2014 ↗ 2020		100	2A	2001–2010 = 2020	
Slovenia	100	3/2	1	2008–2017 ↗ 2020	Cervical and corpus uteri cancers estimated using survival (source EUROCARE-5)	100	1	2003–2012 ↗ 2020	
Spain	100	2/2	1	2008–2017 ↗ 2020	Cervical and corpus uteri cancers estimated using survival (source EUROCARE-5)	27	3A	Estimated from mortality with M:I ratios from 14 Spanish cancer registries	Breast, prostate and thyroid cancers from 7 cancer registries (2003–2012 ↗ 2020) Kaposi sarcoma, non-melanoma skin and childhood cancers from 14 cancer registries (2008–2012 = 2020)
Sweden	100	3/4	1	2008–2017 ↗ 2020	Cervical and corpus uteri cancers estimated using survival (source NORDCAN)	100	1	2007–2016 ↗ 2020	Anus, vulva, vagina, non-melanoma skin and penile cancers, mesothelioma, Kaposi sarcoma (not available in NORDCAN) and brain and central nervous system tumours from Swedish Cancer Registry. Liver and pancreatic cancer cases completed by cancer deaths (source WHO).
Switzerland	100	5/4	1	2007–2016 ↗ 2020	Cervical and corpus uteri cancers estimated using survival (source EUROCARE-5)	62	3A	Estimated from mortality with M:I ratios from 11 Swiss cancer registries	Breast and thyroid cancers estimated from 8 Swiss cancer registries (2003–2012 ↗ 2020) Kaposi sarcoma and Hodgkin lymphoma; nasopharyngeal, anus, vulva, vagina, penile, testis and childhood from 11 cancer registries (2008–2012 = 2020) Non-melanoma skin cancers from 5 cancer registries (2003–2012 ↗ 2020).
Ukraine	93	4/4	1	2008–2017 ↗ 2020	Data of gallbladder, testis, kidney, thyroid cancers and Hodgkin lymphoma plus ‘other cancers’ from National Cancer Registry of Ukraine^f^	100	1	2006–2015 ↗ 2020	
United Kingdom	100	1/3	1	2007–2016 ↗ 2020	Cervical and corpus uteri cancers estimated using survival (source EUROCARE-5)	100	1	2004–2013 ↗ 2020	Non-melanoma skin cancers estimated from Northern Ireland and Scotland (2004–2013 ↗ 2020)

National cancer mortality data from 2004 to 2018 were extracted from WHO mortality database, unless otherwise specified. National and local cancer incidence data from 2003 to 2018 were received from national and local population-based cancer registries, unless otherwise specified.

‘↗’ projected to.

‘=’ applied to.

a Percentages of ill-defined causes of death, most recent year, male/female.

b The method to estimate the national sex- and age-specific mortality rates in 2020 is based on estimated numbers obtained as: 1 - Estimates based on national mortality data, projected rates applied to 2020 population 2A - Estimates based on national mortality data, most recent available rates applied to 2020 population.

c For descriptions of methods applied in these cancer sites, please refer to https://gco.iarc.fr/today/data/methods/GLOBOCAN2020_annexes.pdf [Accessed January 2021].

d The methods to estimate the national sex- and age-specific incidence rates in 2020 are based on estimated numbers obtained as: 1- Estimates based on national or local (coverage greater than 50%) incidence data, projected rates applied to 2020 population 2A - Estimates based on national incidence data, most recent available rates applied to 2020 population 3A - Estimates based on national mortality estimates and M:I ratios derived from country-specific cancer registry data 3B - Estimates based on national mortality estimates and M:I ratios derived from cancer registry data of neighbouring countries.

e Southern Europe model includes Croatia, Cyprus, Italy (33 registries), Malta, Slovenia and Spain (14 registries).

f Data source: National Cancer Registry of Ukraine, National Institute of Cancer. Cancer in Ukraine 2013–2014, 2014–2015 and 2015–2016. Kyiv, Ukraine Available at http://www.unci.org.ua/, accessed 31-08-2017.

g All age incidence partitioned by age using the age distribution from Republika of Srpska.

h Incidence and mortality rates (2007–2016, government-controlled area of Cyprus) were projected to 2020 and applied to 2020 population including both Greek and Turkish parts.

i Eastern Europe model includes Bulgaria, Romania (2 registries) and Serbia.

j 5-year mortality 2012–2016 was used because the number of estimated deaths in 2020 was too small for some sites. The estimated numbers of cases (2012–2016) were then divided by 5.

k Data source: Nouveaux cas de cancer 2013. Registre morphologique des tumeurs. Laboratoire national de santé. Grand-Duché de Luxembourg, 2015 Available as http://www.lns.public.lu/publications/brochures/RMT_Nouveaux_cas_de_cancer_2013.pdf [Accessed 5 January 2017].

l Mortality and Incidence data source: Malignant neoplasms in Montenegro 2013. Podgorica: Insitute of Public Health of Montenegro, Center for Control and Prevention of Non-communicable Diseases, Registry of Malignant Neoplasms of Montenegro, 2018.

m Data source: Russian Federation, Moscow Research Oncological Institute. http://www.oncology.ru [Accessed November 2019].

n Projected population 2020 for Serbia (source UN) included Kosovo.

o Mortality data from WHO excludes Kosovo.

The accuracy of death certificates of cancer of the uterus presents large variations among European countries owing to the fact that many deaths are classified as "uterus cancer, not otherwise specified" (ICD-10 C55) [22]. These cancer deaths were reallocated to either cervix uteri or corpus uteri cancer sites in different ways according to the proportion of unspecified uterus cancers [12]. For countries where the proportion of unspecified uterus cancer was not sufficiently low (defined as <25% of the total), these cancers were distributed using the estimated cancer incidence and the corresponding 5-year relative survival probabilities extracted from the EUROCARE-5 study [23].

For Albania, the Russian Federation and Ukraine, national mortality data for several cancer sites selected for this study were not available and supporting mortality data were used from Croatia and Serbia, from the two Russian cancer registries of St-Petersburg and Arkhangelsk [14], and from the Ukrainian National Cancer Registry [24], respectively.

### 2.2 Estimation methods

The methods used to predict the 2020 estimates depended on the availability and extent of the cancer data as well as the historical trends. The most recent trends in mortality and incidence rates were estimated for the year 2020 using previously applied methodology [12]. When the time series of available historical data were of sufficient duration (at least 6 years), with sufficient numbers of cases or deaths observed for each year and age group, a general approach was to apply short-term time-linear models [25]. This approach was used for data with at least 50 new cancer cases or cancer deaths, for all ages combined, per year. For cancer sites with low incidence, the predictions were obtained assuming fixed sex- and age-specific incidence rates estimated using the most recent 3–5 years of available data to avoid any potential bias resulting from very low numbers. For each cancer site, the predicted age-specific mortality and incidence rates were smoothed by applying moving regression [26] by country and sex in order to remove occasional extreme random variability in the predicted age-specific rates. The applied methodology had been previously verified using real data, and had proved to be robust [[27], [28], [29]]. Statistical computations were carried out using software developed by IARC [11] and JRC [30].

**Fig. 1:**
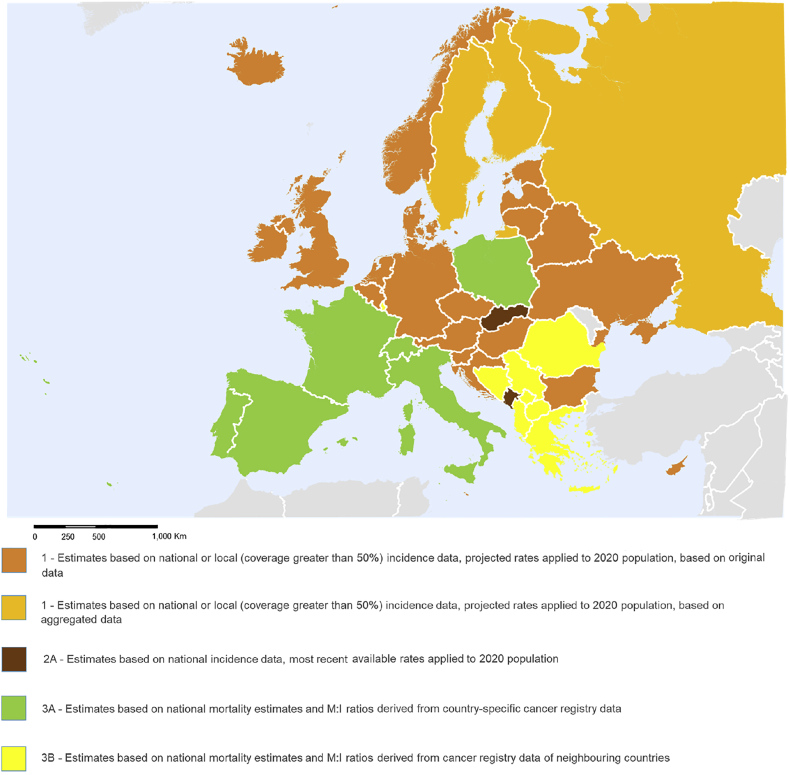
Methods of estimation for 2020 incidence in Europe. © European Union, 2016. Map produced by EC-JRC. The boundaries and the names shown on this map do not imply official endorsement or acceptance by the European Union.

The applied methods are summarised in Table 1 for incidence and mortality, and in Fig. 1 for incidence.

#### 2.2.1 Mortality estimates

Predictions of national mortality rates were performed using the general approach based on short-term prediction models for the majority of the European countries (method 1, applied in 37 countries). In only 3 out of 40 countries, where a sufficient number of deaths were not recorded or where a prediction trend could not be observed, the predictions were performed based on the most recent available data (method 2A, applied in 3 countries).

#### 2.2.2 Incidence estimates

The availability of incidence data is significantly lower compared to mortality data both in terms of length of time series and geographical coverage. The general approach to prediction was adapted and modified depending on the extent and quality [14] of the incidence data available for a given country. The methods applied fall into one of four hierarchical categories described in sections 2.2.2.1, 2.2.2.2, 2.2.2.3, 2.2.2.4.

##### 2.2.2.1 Method 1 – estimates based on national or local incidence data (23 countries)

For the large majority of cases in which countries have either national incidence data or above 60% population coverage, the general prediction method described in section 2.2 was applied.

##### 2.2.2.2 Method 2A – estimates based on most recent incidence national or local incidence data (2 countries)

For too-sparse incidence data in the time interval used as a basis for prediction, the most recent incidence rates were considered, where available, for at least three consecutive years and with at least 20 cases recorded per year. These were then applied to the 2020-projected population figures to obtain the 2020 estimates.

For Slovakia, despite the availability of a long time series, the most recent available rates (2001–2010) were applied to the projected populations to achieve the most robust result (method 2A) for all the cancer sites.

Also for cancer sites where diagnosis could be connected with screening programmes over the period considered as the basis of prediction (including prostate specific antigen [PSA] testing), further examination of the observed and estimated incidence trends was carried out to prevent unrealistic predictions. When the estimates were not judged robust enough (particularly for female breast, thyroid and prostate cancers), predictions were obtained by applying the incidence rate of the otherwise most recent available period to the 2020 population (Table 1).

##### 2.2.2.3 Method 3A – estimates based on local incidence and mortality data (7 countries)

For countries with regional registries, the estimated national sex-, site- and age-specific incidence estimates (I_N_) were obtained using corresponding aggregated regional incidence (I_R_) and national mortality (M_N_) estimates. Under the assumption that regional incidence to mortality ratios are similar to the national ones we derive the formula:

$${\text{I}}_{\text{N}}={\text{M}}_{\text{N}}^{}\text{∗}\left({\text{I}}_{\text{R}}/{\text{M}}_{\text{R}}\right).$$

When dealing with regional registries, aggregated mortality to incidence ratios were calculated using a weighted average over the registries, with weights corresponding to the square roots of the registries’ populations to guarantee the representativeness of the aggregation. A Poisson regression model was then applied to obtain the I_R_/M_R_ ratios, with categorical terms for sex and age.

This method produces unbiased estimates under the assumptions that regional and national incidence to mortality ratios can be considered equivalent and that I_R_/M_R_ ratios are stable over recent time. In those cases where such assumptions do not hold (e.g. for screening-dependent cancer sites), the most recent available incidence rates from the aggregated data were used as a proxy for the national predictions.

##### 2.2.2.4 Method 3B – estimates based on no incidence data and national mortality data (8 countries)

For cases where incidence data either (a) were not available; (b) covered only a small area; or (c) were judged of poor quality [14], the I_R_/M_R_ ratios were obtained from neighbouring countries. The estimates were computed using as reference either Eastern Europe countries, i.e. Bulgaria, Romania (2 registries) and Serbia, or Southern Europe countries, considering Croatia, Cyprus, Italy (33 registries), Malta, Slovenia and Spain (14 registries).

For Luxembourg, the data of 16 cancer registries in Belgium and France were considered for the computation of the neighbouring I_R_/M_R_ ratios applied to the 5-year national mortality data (2012–2016). Such results were then completed using the data of the Luxembourg pathology register [23] according to the procedure previously applied [12].

### 2.3 Estimates presentation

The estimates for 2020 are presented as numbers of new cases and numbers of deaths and corresponding age-standardised rates (ASRs) for incidence and mortality using the European standard population 2013 [31,32] expressed per 100,000 individuals. The cumulative risk of a cancer diagnosis or cancer death was also calculated, considering a lifetime as across the age range 0–74 years, expressed as a percentage. To quantify the variability between countries, the relative difference between the country-specific ASR and the European average as the reference ASR. The relative difference is expressed as a percentage.

## 3 Results

The numbers of new cancer diagnoses and cancer deaths estimated to occur in Europe for 2020 (in thousands) by cancer type and sex are reported in Fig. 1 and Table 2. The estimated cancer burden in Europe for 2020 is 4.0 million new cancer cases (all cancer types, excluding non-melanoma skin cancer) and 1.9 million cancer deaths. The corresponding cumulative risk (below 75 years) of receiving a cancer diagnosis is 31% for males (1 in 3 men) and 24% for females (1 in 4 women), while the corresponding risk of dying due to cancer is 15% for males (1 in 7 men) and 9% for females (1 in 11 women). Cancer overall affect men slightly more than women, with 53% (2.1 million) of new cases and 55% (1.1 million) of cancer deaths occurring in males. Cancer disproportionately affects older adults, with 60% of the estimated new diagnoses and 73% of estimated deaths occurring in persons aged 65 years or older, 34% and 25% occurring in people 45–64 years old, and 7% and 3% in people younger than 45 years.

**Table 2:** Estimated numbers of new cancer cases and deaths from cancer (thousands), ASRs^a^ (per 100,000) by sex and cancer site in Europe for 2020.

Cancer site	Incidence												Mortality											
	Both sexes				Male				Female				Both sexes				Male				Female			
	Cases	%	ASR (E)	Cum. Risk %	Cases	%	ASR (E)	Cum. Risk %	Cases	%	ASR (E)	Cum. Risk %	Deaths	%	ASR (E)	Cum. Risk %	Deaths	%	ASR (E)	Cum. Risk %	Deaths	%	ASR (E)	Cum. Risk %
Lip, oral cavity and pharynx	128.6	3.2	16.9	1.11	93.3	4.4	27.2	1.81	35.3	1.8	8.4	0.50	53.9	2.8	7.1	0.44	41.4	3.8	12.3	0.78	12.5	1.4	2.9	0.15
Oesophagus	53.0	1.3	7.0	0.41	40.4	1.9	12.3	0.72	12.6	0.7	2.9	0.15	45.5	2.3	6.1	0.33	34.9	3.2	10.8	0.59	10.6	1.2	2.4	0.11
Stomach	136.0	3.4	18.1	0.96	83.7	3.9	26.2	1.39	52.3	2.7	12.1	0.61	97.0	5.0	12.9	0.63	59.5	5.5	18.9	0.93	37.5	4.3	8.6	0.39
Colorectum	519.8	12.9	69.4	3.60	281.7	13.2	89.0	4.50	238.1	12.5	55.4	2.85	244.8	12.6	32.9	1.36	131.9	12.3	43.5	1.77	112.9	13.0	25.5	1.02
Liver	87.6	2.2	11.7	0.63	58.1	2.7	18.0	0.99	29.6	1.5	6.8	0.32	78.4	4.0	10.5	0.52	51.3	4.8	16.3	0.80	27.1	3.1	6.2	0.27
Gallbladder	12.6	0.3	1.7	0.08	3.9	0.2	1.3	0.06	8.7	0.5	2.0	0.09	8.7	0.4	1.2	0.05	2.6	0.2	0.9	0.03	6.1	0.7	1.4	0.06
Pancreas	140.1	3.5	18.7	0.91	70.2	3.3	22.2	1.13	69.9	3.7	16.0	0.73	132.1	6.8	17.7	0.84	66.7	6.2	21.2	1.05	65.4	7.6	14.9	0.67
Larynx	39.9	1.0	5.2	0.36	35.0	1.6	10.3	0.68	4.9	0.3	1.2	0.08	19.6	1.0	2.6	0.16	17.6	1.6	5.4	0.32	2.0	0.2	0.5	0.03
Lung	477.5	11.8	63.5	3.70	315.1	14.8	97.6	5.40	162.5	8.5	38.3	2.26	384.2	19.8	51.2	2.82	260.0	24.2	81.7	4.27	124.2	14.3	29.0	1.60
Melanoma of skin	150.6	3.7	20.0	1.22	76.3	3.6	23.2	1.31	74.3	3.9	18.2	1.16	26.4	1.4	3.5	0.17	14.7	1.4	4.7	0.22	11.7	1.3	2.7	0.13
Breast									531.1	27.8	128.6	8.01									141.8	16.4	32.9	1.62
Cervix uteri									58.2	3.0	14.6	1.03									26.0	3.0	6.3	0.40
Corpus uteri									130.1	6.8	31.2	2.05									30.0	3.5	6.9	0.35
Ovary									66.7	3.5	16.0	1.01									44.1	5.1	10.3	0.58
Prostate					473.3	22.2	148.1	8.18									108.1	10.0	38.2	0.97				
Testis					25.1	1.2	6.7	0.51									1.6	0.1	0.4	0.03				
Kidney	138.6	3.4	18.4	1.11	85.8	4.0	25.9	1.54	52.8	2.8	12.5	0.74	54.1	2.8	7.2	0.34	34.6	3.2	11.1	0.52	19.5	2.2	4.4	0.19
Bladder	204.0	5.0	27.3	1.36	156.7	7.4	50.5	2.35	47.3	2.5	10.9	0.54	67.3	3.5	9.1	0.31	50.8	4.7	17.5	0.54	16.5	1.9	3.6	0.11
Brain, central nervous system	67.1	1.7	8.9	0.57	36.2	1.7	10.6	0.68	30.9	1.6	7.5	0.48	53.7	2.8	7.1	0.44	29.4	2.7	8.8	0.53	24.3	2.8	5.8	0.35
Thyroid	87.2	2.2	11.5	0.83	19.3	0.9	5.5	0.38	67.8	3.5	17.1	1.24	6.4	0.3	0.9	0.04	2.4	0.2	0.8	0.04	4.0	0.5	0.9	0.04
Hodgkin lymphoma	19.9	0.5	2.7	0.19	11.0	0.5	3.1	0.22	8.9	0.5	2.3	0.17	4.0	0.2	0.5	0.03	2.3	0.2	0.7	0.04	1.7	0.2	0.4	0.02
Non-Hodgkin lymphoma	123.0	3.0	16.4	0.92	67.4	3.2	20.7	1.13	55.6	2.9	13.1	0.74	49.7	2.6	6.7	0.28	27.2	2.5	9.0	0.36	22.5	2.6	5.1	0.20
Multiple myeloma	50.9	1.3	6.8	0.36	27.8	1.3	8.9	0.44	23.1	1.2	5.4	0.29	32.5	1.7	4.4	0.18	17.1	1.6	5.7	0.22	15.4	1.8	3.5	0.15
Leukaemia	100.0	2.5	13.4	0.73	55.8	2.6	17.4	0.91	44.2	2.3	10.5	0.58	62.3	3.2	8.4	0.35	34.3	3.2	11.4	0.45	27.9	3.2	6.4	0.27
All sites excl. non-melanoma skin	4042.3		538.0	27.02	2130.2		660.3	30.83	1912.1		456.1	23.95	1942.6		259.7	11.66	1076.6		347.8	14.66	866.0		199.5	9.11

a ASR = Age-standardised rates using European standard population 2013, based on estimated number of new cases or deaths.

**Fig. 2:**
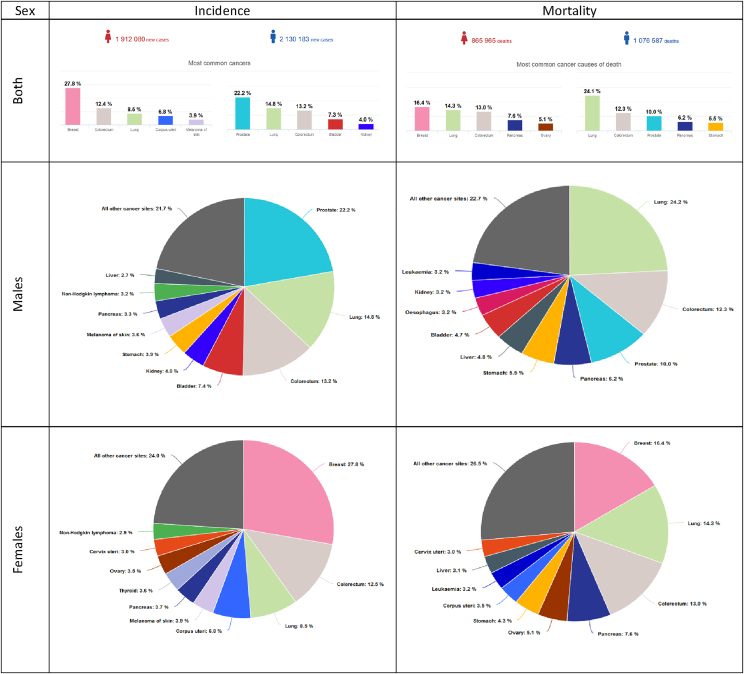
Total number and distribution of the estimated cases and deaths for the 5 (in the bar charts) or 10 (in the pie charts) most common cancers in Europe for 2020 by sex. For each sex, the area of the bars and pie charts reflects the proportion of the total number of cases or deaths.

Four cancer types are responsible for approximately 50% of all cancer diagnoses. Breast cancer is the most commonly diagnosed cancer accounting for 13.1% of all cancer diagnoses (530,000 cases; females only), followed by colorectal (520,000, 12.9%), lung (480,000, 11.8%) and prostate (470,000, 11.7%) cancers. When distinguishing by sex (Fig. 2), the most common cancers among males are prostate (22.2% of the male total), lung (320,000, 14.8%), colorectum (280,000, 13.2%) and bladder (160,000, 7.3%). In women, breast cancer is by far the most frequently diagnosed malignant neoplasm (27.8% of the female total), followed by colorectal (240,000, 12.4%), lung (160,000, 8.5%) and corpus uteri (130,000, 6.8%) cancers.

For mortality, the most common causes of cancer death are due to lung (380,000 deaths, corresponding to one-fifth of the overall share), colorectal (250,000 deaths, 12.6%), breast (140,000, 7.3%; females only) and pancreatic (130,000, 6.8%) cancers. All together, these cancers account for 47% of all-cancers mortality. When considering the male population only (Fig. 2), lung cancer is the most common cause of cancer-related death (260,000 estimated deaths, corresponding to 24.2% of the male total) followed by colorectal (130,000, 12.3%) and prostate (110,000, 10.0%) cancers. For females, breast cancer is the leading cause of cancer death (140,000, 16.4% of female total), followed by lung (120,000, 14.3%) and colorectal (110,000, 13.0%) cancers. In Table 3, the leading three types of cancer for incidence and mortality are reported by European country, for males and females separately.

**Table 3:** Most common types of cancer in terms of new cases (incidence) and deaths (mortality) in each of the European countries for 2020.

Focussing on the EU-27 (Table 4), the cancer burden in 2020 corresponds to 2.7 million new cases and 1.3 million deaths, representing 66% of all new cases and 65% of cancer deaths of the whole of Europe. The overall cumulative risk of cancer diagnosis and deaths are very similar to European figures. Breast cancer is the most commonly diagnosed cancer with over 360,000 new female cases (13.3% of all cancer diagnoses), followed by colorectal (340,000, 12.7%), prostate (340,000, 12.5%) and lung (320,000, 11.9%) cancers. The most common causes of cancer death are lung (260,000 deaths, 20.4% of all deaths), colorectal (160,000, 12.4%), breast (90,000, 7.3%; females only) and pancreatic (90,000, 7.1%) cancers.

**Table 4:** Estimated numbers of new cancer cases and deaths from cancer (thousands), ASRs^a^ (per 100,000) by sex and cancer site in EU-27 for 2020.

Cancer site	Incidence												Mortality											
	Both sexes				Males				Females				Both sexes				Males				Females			
	Cases	%	ASR (E)	Cum. Risk %	Cases	%	ASR (E)	Cum. Risk %	Cases	%	ASR (E)	Cum. Risk %	Deaths	%	ASR (E)	Cum. Risk %	Deaths	%	ASR (E)	Cum. Risk %	Deaths	%	ASR (E)	Cum. Risk %
Lip, oral cavity and pharynx	79.9	3.0	17.0	1.11	56.0	3.9	25.6	1.69	23.9	1.9	9.3	0.56	31.8	2.5	6.7	0.40	23.8	3.4	11.0	0.68	8.0	1.4	3.0	0.15
Oesophagus	30.3	1.1	6.4	0.38	23.3	1.6	10.9	0.65	7.0	0.6	2.7	0.15	25.6	2.0	5.4	0.29	19.9	2.8	9.4	0.51	5.8	1.0	2.1	0.10
Stomach	75.4	2.8	15.8	0.78	46.7	3.2	22.4	1.10	28.8	2.3	10.6	0.49	52.1	4.1	10.9	0.48	32.1	4.6	15.7	0.69	19.9	3.6	7.2	0.30
Colorectum	341.4	12.7	71.8	3.65	191.1	13.2	91.6	4.58	150.4	12.2	56.3	2.82	156.1	12.4	32.4	1.27	87.2	12.3	43.1	1.67	68.9	12.4	24.5	0.91
Liver	60.9	2.3	12.8	0.70	41.9	2.9	19.8	1.11	19.0	1.5	7.0	0.32	53.9	4.3	11.3	0.55	36.6	5.2	17.6	0.86	17.3	3.1	6.3	0.27
Gallbladder	7.8	0.3	1.6	0.07	2.7	0.2	1.3	0.06	5.1	0.4	1.9	0.09	5.5	0.4	1.1	0.05	1.8	0.3	0.9	0.04	3.7	0.7	1.3	0.06
Pancreas	94.9	3.5	19.9	0.94	47.1	3.3	22.6	1.12	47.8	3.9	17.5	0.78	89.3	7.1	18.6	0.86	44.7	6.3	21.6	1.03	44.5	8.0	16.2	0.70
Larynx	24.6	0.9	5.2	0.35	21.2	1.5	9.8	0.63	3.5	0.3	1.4	0.09	11.6	0.9	2.4	0.14	10.2	1.4	4.8	0.27	1.3	0.2	0.5	0.03
Lung	318.3	11.9	67.3	3.93	205.3	14.2	97.2	5.30	113.1	9.1	43.9	2.68	257.3	20.4	54.2	2.97	170.6	24.2	81.7	4.14	86.7	15.6	33.2	1.91
Melanoma of skin	106.4	4.0	22.8	1.41	55.4	3.8	26.0	1.50	51.0	4.1	20.8	1.35	16.5	1.3	3.5	0.16	9.5	1.3	4.6	0.21	7.0	1.3	2.6	0.12
Breast									355.5	28.7	142.8	8.85									91.8	16.5	34.1	1.60
Cervix uteri									30.4	2.5	12.8	0.89									13.4	2.4	5.3	0.32
Corpus uteri									73.3	5.9	28.9	1.86									16.8	3.0	6.2	0.29
Ovary									39.4	3.2	15.5	0.94									27.1	4.9	10.3	0.55
Prostate					335.5	23.2	158.7	8.95									69.9	9.9	36.3	0.82				
Testis					17.6	1.2	8.0	0.61									0.9	0.1	0.4	0.03				
Kidney	86.8	3.2	18.4	1.09	55.2	3.8	25.8	1.52	31.6	2.6	12.1	0.70	34.4	2.7	7.2	0.32	22.2	3.1	10.8	0.47	12.2	2.2	4.4	0.18
Bladder	157.5	5.9	33.1	1.65	121.5	8.4	58.9	2.73	35.9	2.9	13.4	0.67	49.2	3.9	10.1	0.33	37.1	5.3	18.8	0.56	12.0	2.2	4.2	0.13
Brain, central nervous system	43.6	1.6	9.4	0.59	23.8	1.7	11.1	0.71	19.7	1.6	7.8	0.49	34.7	2.8	7.4	0.45	19.4	2.7	9.0	0.55	15.3	2.8	6.0	0.35
Thyroid	57.7	2.2	12.7	0.91	13.7	0.9	6.2	0.44	44.0	3.6	18.9	1.37	4.0	0.3	0.8	0.03	1.6	0.2	0.8	0.03	2.4	0.4	0.9	0.03
Hodgkin lymphoma	12.0	0.4	2.7	0.19	6.9	0.5	3.2	0.22	5.1	0.4	2.2	0.16	2.2	0.2	0.5	0.02	1.3	0.2	0.6	0.03	0.9	0.2	0.3	0.02
Non-Hodgkin lymphoma	86.3	3.2	18.3	1.03	47.9	3.3	22.6	1.25	38.5	3.1	14.8	0.82	34.9	2.8	7.3	0.29	19.3	2.7	9.5	0.37	15.6	2.8	5.6	0.21
Multiple myeloma	35.8	1.3	7.5	0.39	20.1	1.4	9.7	0.48	15.7	1.3	5.9	0.30	23.3	1.8	4.8	0.19	12.5	1.8	6.2	0.23	10.8	1.9	3.9	0.15
Leukaemia	66.6	2.5	14.1	0.75	37.6	2.6	18.0	0.93	29.0	2.3	11.1	0.59	43.4	3.4	9.0	0.35	24.2	3.4	12.0	0.45	19.2	3.5	6.9	0.27
All sites excl. non-melanoma skin	2682.0		568.7	28.20	1444.9		685.2	31.73	1237.0		483.6	25.07	1261.7		263.5	11.38	706.1		344.9	13.94	555.6		204.5	9.05

a ASR = Age-standardised rates using European standard population 2013, based on estimated number of new cases or deaths.

**Fig. 3:**
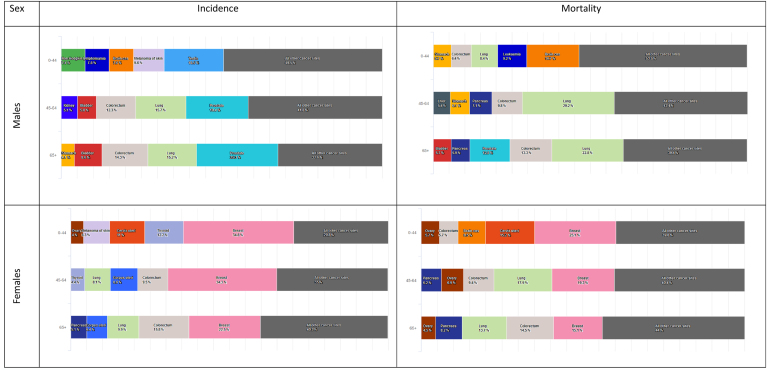
Cancer incidence and mortality percentage distribution by age group and sex in Europe for 2020.

Age-stratified analyses, relating to three different age groups (0–44, 45–64 and 65 years and over, Fig. 3), revealed that the five most common cancers for incidence and mortality predominantly affect middle-aged and older adults. In the 0–44 years group, more than 50% of incident cases are due to breast, thyroid, and cervical cancer among females, and to testicular cancer, melanoma of the skin, brain and other CNS, leukaemia and non-Hodgkin lymphoma among males. In older age groups, the most commonly diagnosed cancer among females is breast (34.5% in 45–64 and 22.5% in 65+), followed by colorectal (9.5% and 15.8%), corpus uteri (8.6% and 6.4%), lung (8.1% and 9.9%), thyroid (4.4% in the 45–64 age groups) and pancreatic (5.1% in the 65+ age group) cancers. Among males, prostate cancer is the most commonly diagnosed (19.4% in 45–64 and 25.3% in 65+), followed by lung (15.7% and 15.2%), colorectal (12.3% and 14.3%), and bladder (5.8% and 8.6%) cancers. The fifth most common cancer in males is kidney cancer in the 45–64 years group (5.1%) and stomach cancer (4.1%) in the 65+ years group.

Similar to incidence, younger age groups have different mortality distributions as compared to middle and older ages (Fig. 3). Among males, lung cancer accounts for the highest number of cancer-related deaths (29.2% in 45–64 and 22.8% in 65+), followed by colorectal (9.8% and 13.3%) and pancreatic (7.1% and 5.9%) cancers. Liver (5.4%) and stomach (6.1%) cancers are the main causes of cancer deaths in the age class 45–64 years, while prostate (12.8%) and bladder (5.7%) cancers rank highest in the older age classes. Among females, breast cancer has the highest proportion of cancer deaths (19.3% in 45–64 and 15.1% in 65+ years), followed by lung (17.9% and 13.7%), colorectal (9.4% and 14.5%), ovary (6.9% and 4.5%), and pancreatic (6.2% and 8.2%) cancers.

For the 40 European countries analysed, Appendix Table A1a, Appendix Table A1b present the estimated number of new cases and the incidence rates by sex and site, while Appendix Table A2a, Appendix Table A2b present the estimated number of deaths and mortality rates by sex and site. Appendix Table A3, Appendix Table A4 report the estimated number of new cases and deaths with the incidence and mortality rates by age group and site.

Sections 3.1, 3.2, 3.3, 3.4, 3.5, 3.6 provide a brief description of the overall cancer patterns and highlight the five cancers that are the most common cancer diagnoses or main causes of cancer-related deaths in Europe.

### 3.1 Overall cancer patterns

After adjusting for different population age structures, overall cancer incidence rates are highest: (a) in Ireland for both sexes combined (718.3 per 100,000, +33% as compared to the European average, data not shown); (b) in Latvia when considering only male population (851.7, +28%); and (c) in Denmark for female population (633.9, +38%).

**Fig. 4:**
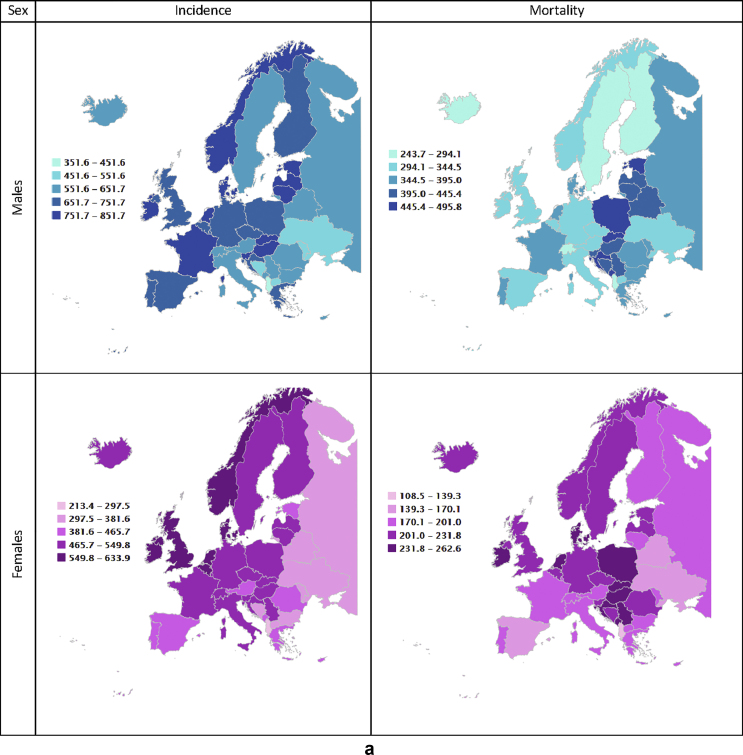

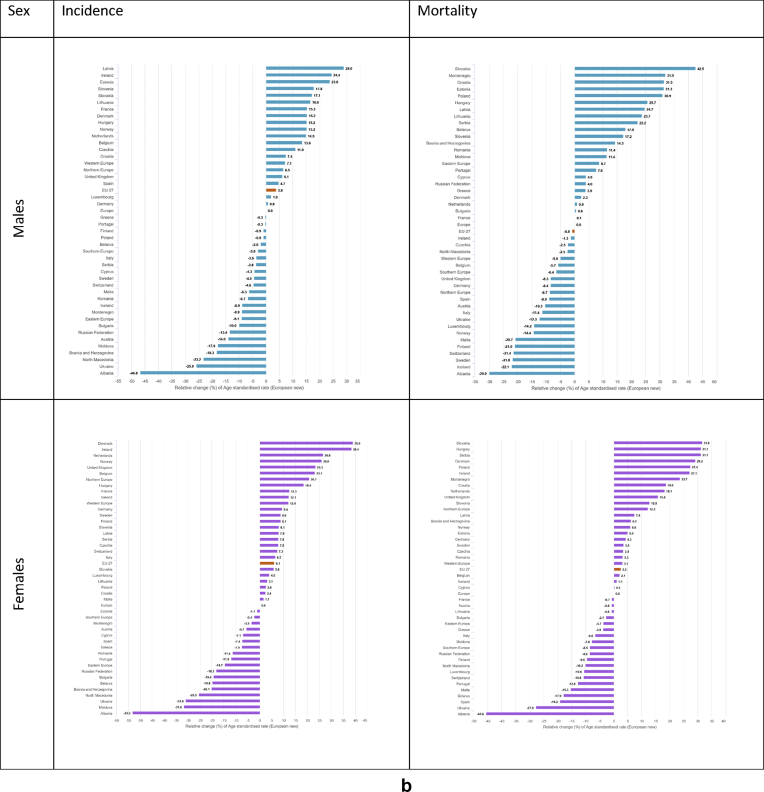
a: Age-standardised rates∗ (per 100,000) for all cancers excluding non-melanoma skin cancers by country and sex in Europe for 2020. b: Relative difference (percent) in age-standardised incidence and mortality rates for all cancers excluding non-melanoma skin cancers by country and sex in Europe for 2020, using the European average rate as reference. ∗European standard population 2013, based on estimated number of new cases or deaths.

**Fig. 5:**
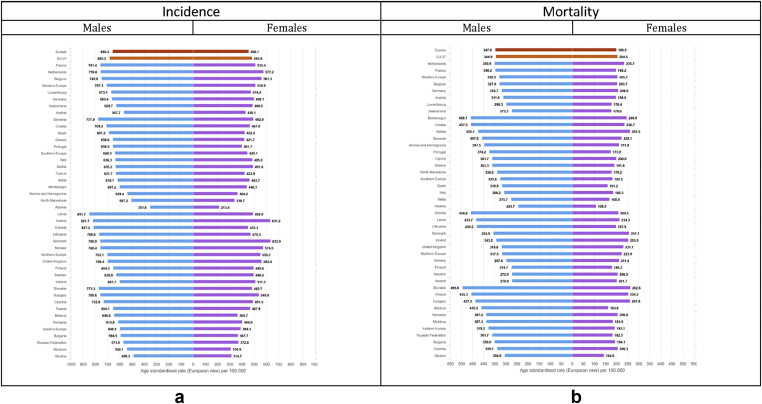
(a) Age-standardised incidence rates∗ (per 100,000) by sex, area and country in Europe for 2020 for all cancers excluding non-melanoma skin cancers. (b) Age-standardised mortality rates∗ (per 100,000) by sex, area and country in Europe for 2020 for all cancers excluding non-melanoma skin cancers. ∗European standard population 2013, based on estimated number of new cases or deaths.

For males, the highest incidence rates above 750 per 100,000 (only 20% of countries reported incidence rates above this threshold – last quintile) occur in several countries (Latvia, Ireland, Estonia, Slovenia, Slovakia, Lithuania, France, Denmark, Hungary, Norway, and the Netherlands, Fig. 4, Fig. 5), where rates range from 14% to 28% higher than the European average. For females, incidence rates in the last quintile (above 550 per 100,000) occur in Denmark, Ireland, the Netherlands, Norway, United Kingdom and Belgium, where they range from 22% to 38% higher than the European average. The lowest all-cancer incidence rates for both sexes occur in Albania (351.6 per 100,000, −47% as compared to the European average in men, and 213.4, −53% in women), Ukraine (489.3, −26% and 314.7, −31%) and Moldova (542.1, −19% and 310.9, −32%).

The highest cancer mortality rates both for men and women occur in Slovakia (495.8 per 100,000, 41% higher than the European average for males, and 262.6 per 100,000, 31% for females), and high rates were estimated for Eastern and Balkan countries overall (e.g. Montenegro, Poland, Serbia, Hungary, and Croatia). The lowest mortality rates occur in Sweden (272, −23%), Iceland (270.9, −23%), and Albania (243.7, −31%) in the male population, and for Spain (161.2, −20%), Ukraine (144, −28%) and Albania (108.5, −46%) in the female population (Fig. 4, Fig. 5).

### 3.2 Breast cancer in females

Breast cancer was estimated to be the most frequently diagnosed cancer among women in all European countries (Table 3 and Fig. 2), and the first cause of female cancer mortality in the majority of countries [24 (60%) European countries and 14 (52%) EU countries]. The cumulative risk of a breast cancer diagnosis before the age of 75 is 8% (1 in 12 women) while the risk of breast cancer death before the age of 75 is 1.6% (1 in 61 women) in Europe. Similar values were estimated for EU-27 where breast cancer was estimated to be the most commonly diagnosed cancer in all age groups (35% of cases in women 0–44 years, 34% for 45–64 years, and 23% in women 65 years or older), and the first cause of cancer mortality in each age group (25%, 19%, 15% respectively).

**Fig. 6:**
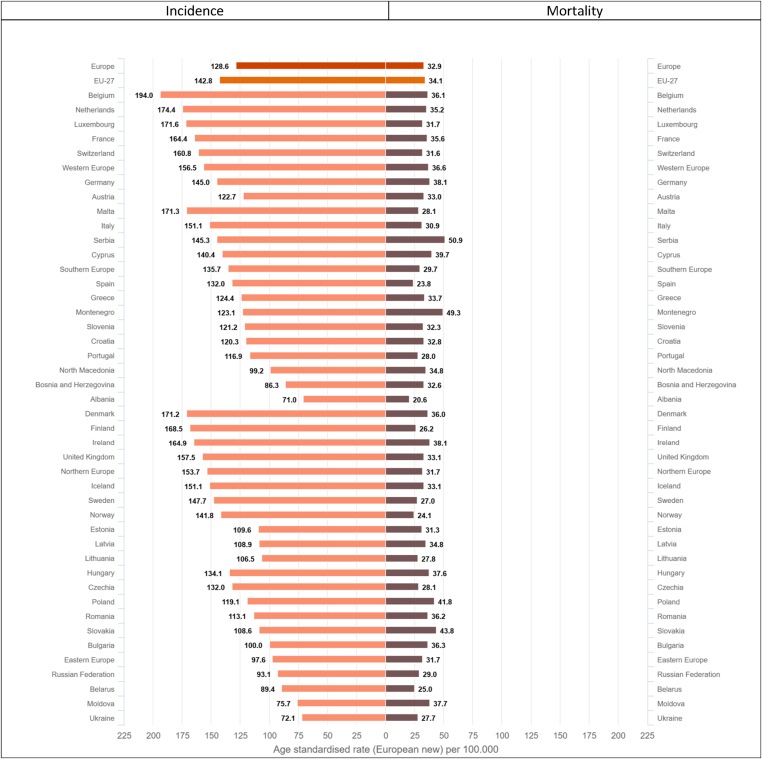
Age-standardised incidence and mortality rates∗ (per 100,000) by area and country in Europe for 2020 for breast cancer. ∗European standard population 2013, based on estimated number of new cases or deaths.

There are large variations in the estimated incidence rates of breast cancer among European countries (almost threefold from 71 to 194 per 100,000, Fig. 6). High incidence rates were estimated for countries in Western Europe–Belgium (ASR of 194 per 100,000), the Netherlands (174.4), and Luxembourg (171.6), in Northern Europe–Denmark (171.2), Finland (168.5), and Ireland (164.9), as well as in Southern Europe–Malta (171.3). Incidence rates are considerably lower in Eastern Europe and Balkan countries including Bosnia and Herzegovina (86.3), Moldova (75.7), Ukraine (72.1), and Albania (71) (Fig. 6).

The range of mortality rates also vary more than twofold (from 20.6 to 50.9 per 100,000), with the highest rates estimated in some countries of Southern and Eastern Europe such as Serbia (50.9 per 100,000), Montenegro (49.3), Slovakia (43.8) and Poland (41.8). The lowest mortality rates (below 25 per 100,000) are found in Southern Europe, notably in Albania (20.6) and Spain (23.8), and in Norway (24.1) (Fig. 6).

### 3.3 Colorectal cancer

**Fig. 7:**
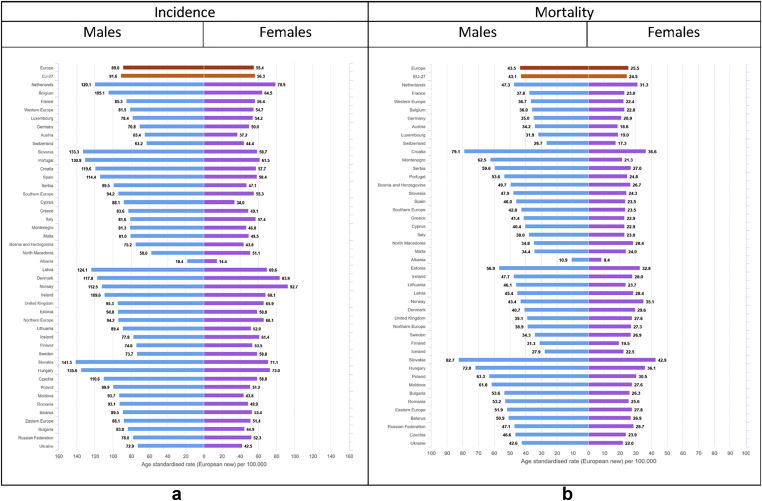
(a) Age-standardised incidence rates∗ (per 100,000) by sex, area and country in Europe for 2020 for colorectal cancer. (b) Age-standardised mortality rates∗ (per 100,000) by sex, area and country in Europe for 2020 for colorectal cancer. ∗European standard population 2013, based on estimated number of new cases or deaths.

Colorectal cancer was estimated to be the second most diagnosed cancer in Europe considering both sexes together. Incidence rates of colorectal cancer are higher in men than in women (European average ASR 89 for males and 55 for females, Fig. 7a). The same is true for the cumulative risk before the age of 75 (4.5% for males, corresponding to 1 in 22 men, and 2.9% for females, corresponding to 1 in 35 women) (Table 2). For males, elevated incidence rates were estimated for Slovakia (141.3 per 100,000), Hungary (135.6) and Slovenia (133.3); among females, the highest rates are observed in Norway (92.7), Denmark (83.9) and the Netherlands (78.9). Geographical differences in incidence rates are notable for both sexes (almost eightfold for males, and almost sevenfold for females), with much lower rates estimated for Albania (18.4 for males, 14.4 for females) than for any other European country (Fig. 7a).

Colorectal cancer is also the second leading cause of cancer death for both sexes (Fig. 2). Geographical patterns of mortality partially follow incidence, the few exceptions are countries with high rates of colorectal cancer mortality compared with relatively low incidence (e.g. Poland) (Fig. 7b).

### 3.4 Lung cancer

There are almost 480,000 new cases of lung cancer (11.8% of all new diagnoses) and more than 380,000 deaths, corresponding to almost 20% of all cancer deaths (Table 2). Lung cancer represents the first cause of cancer mortality among males in all European countries apart from Sweden, and among females in 13 countries (one-third) of the European countries (Table 3 and Fig. 2). Lung cancer affects men more than women, with a male-to-female incidence ratio ranging from 3 to 10. The cumulative risk of lung cancer diagnosis before the age of 75 is also higher for males than for females (5.4% for males corresponding to 1 in 19 men, and 2.3% for females corresponding to 1 in 44 women) (Table 2). Similar values were estimated in EU-27 for males while cumulative risk for females is slightly higher (2.7%, corresponding to 1 in 37 women) (Table 4). Incidence rates of lung cancer among European countries vary threefold in males and almost ninefold in females (Fig. 9). For males, incidence is highest in Central and Eastern Europe–Hungary (138.3 per 100,000), Serbia (136.4), Bosnia and Herzegovina (131.3), and Latvia (127.9), and in some countries of Southern and Western Europe such as Greece (127.2), Montenegro (123.8), and Belgium (123.5). Low rates were estimated for Finland (67.1), Switzerland (64.3), and Sweden (44.8) (Fig. 9a). Among females, the highest rates are seen in Ireland (85.1), Denmark (85.1), Hungary (76.6), Iceland (74.3) and United Kingdom (71.4); the lowest rates are in Eastern Europe, notably Ukraine (11.8) and Belarus (10) (Fig. 9a). Given the relatively poor prognosis of the disease after diagnosis, geographical patterns of mortality rates are quite similar to those of incidence, in both sexes (Fig. 9b).

### 3.5 Prostate cancer

**Fig. 8:**
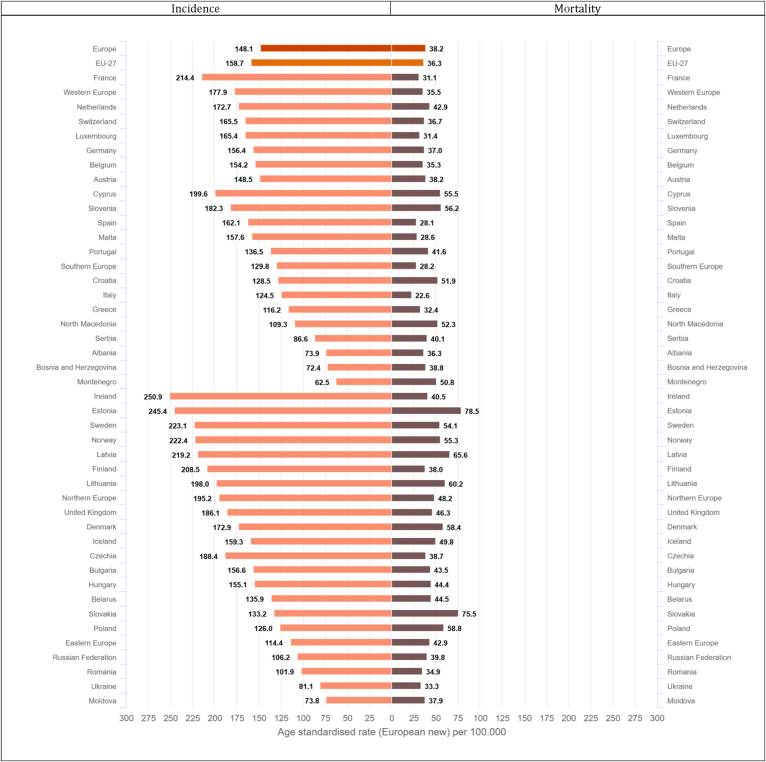
Age-standardised incidence and mortality rates∗ (per 100,000) by area and country in Europe for 2020 for prostate cancer. ∗European standard population 2013, based on estimated number of new cases or deaths.

Prostate cancer was estimated as the fourth most common cancer in Europe in 2020, and is by far the most frequent cancer among males (Fig. 2). The cumulative risk of being diagnosed with prostate cancer before the age of 75 is 8.2% (1 in 12 men), while the risk of prostate cancer death before the age of 75 is1% (1 in 103 men) in Europe. Similar values were estimated also for EU-27 (Table 2, Table 4). Incidence rates of prostate cancer vary fourfold (from 63 per 100,000 of Montenegro to 251 of Ireland). The highest incidence rates were estimated mostly in selected Northern and Western European countries: Ireland (250.9), Estonia (245.4), Sweden (223.1), Norway (222.4), Latvia (219.2), but also France (214.4) and Cyprus (199.6); rates are lowest in Eastern Europe: in Montenegro (62.5), Bosnia and Herzegovina (72.4), Moldova (73.9), Albania (73.9), Ukraine (81.1), and Serbia (86.6) (Fig. 8). Compared to incidence, mortality rates vary less, ranging from the elevated figures estimated for Estonia (78.5) and Slovakia (75.5) to relatively low figures estimated for Spain (28.1) and Italy (22.6) (Fig. 8).

**Fig. 9:**
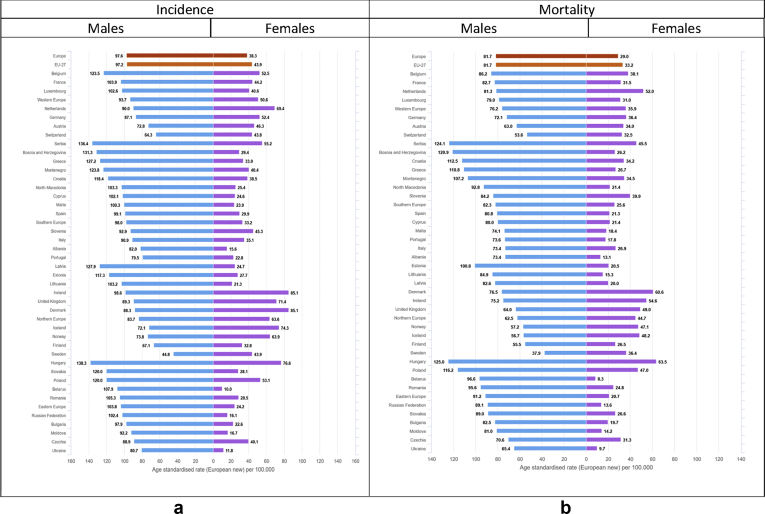
(a) Age-standardised incidence rates∗ (per 100,000) by sex, area and country in Europe for 2020 for lung cancer. (b) Age-standardised mortality rates∗ (per 100,000) by sex, area and country in Europe for 2020 for lung cancer. ∗European standard population 2013, based on estimated number of new cases or deaths.

### 3.6 Pancreatic cancer

**Fig. 10:**
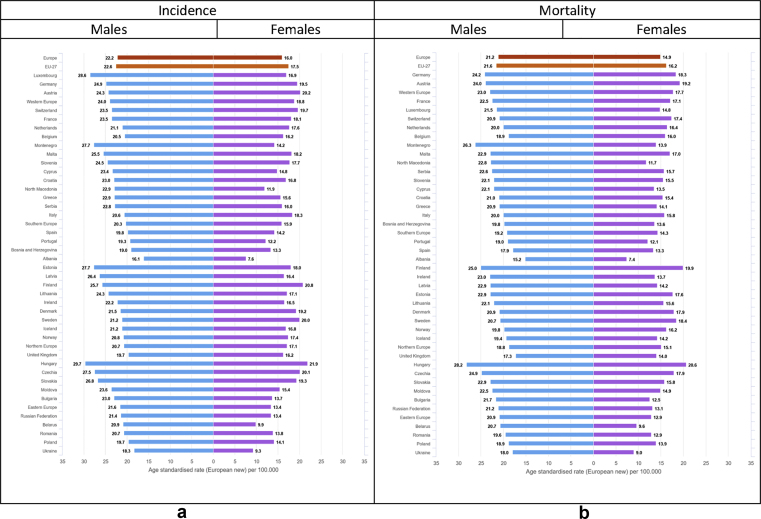
(a) Age-standardised incidence rates∗ (per 100,000) by sex, area and country in Europe for 2020 for pancreatic cancer. (b) Age-standardised mortality rates∗ (per 100,000) by sex, area and country in Europe for 2020 for pancreatic cancer. ∗European standard population 2013, based on estimated number of new cases or deaths.

Pancreatic cancer was estimated to be the fourth leading cause of cancer deaths for both sexes (Fig. 2). There are approximately 140,000 new cases and almost the same number of deaths (Table 2). Pancreatic cancer affects males marginally more than females, with sex ratios closer to 2 mainly in Eastern Europe. The cumulative risk of diagnosis before the age of 75 is similar across sexes, but slightly higher in males (1.1%; 1 in 89 men) than in females (0.7%; 1 in 136 women) (Table 2). Similar values were estimated for EU-27 (Table 4). Rates of pancreatic cancer vary less than twofold among males and almost threefold among females across European countries for both incidence and mortality. For males, high incidence rates are observed in Hungary (29.7 per 100,000 men), Luxembourg (28.6), Estonia (27.7) and Montenegro (27.7), while the lowest incidence was estimated for Spain (19.8), United Kingdom (19.7), Poland (19.7), Portugal (19.3), Bosnia and Herzegovina (19), Ukraine (18.3) and Albania (16.1) (Fig. 10a). For females, a high incidence was estimated for Hungary (21.9), Finland (20.8), Austria (20.2), Czechia (20.1), Sweden (20), and a low incidence for Bosnia and Herzegovina (13.3), Portugal (12.2), North Macedonia (11.8), Belarus (9.9), Ukraine (9.3) and Albania (7.6) (Fig. 10a). Similar patterns are seen in terms of mortality (Fig. 10b).

## 4 Discussion

We estimate over 4 million new cancer cases (excluding non-melanoma skin cancers) and close to 2 million deaths from cancer in Europe in 2020, indicating that on average 11,000 new cancer cases and 5000 cancer deaths occurred every day in Europe over the year. Half of the overall cancer burden in Europe is attributed to breast, colorectal, prostate and lung cancers. The same diseases were also the major causes of cancer deaths in Europe for 2020, but additionally, pancreatic cancer ranks as the fourth leading cause of cancer mortality.

The 2020 estimates still show considerable geographic variability in the incidence and mortality rates among European countries [2] and reflect a number of possible determinants. These include geographic differences in the prevalence of key risk factors for specific cancers, in the effective delivery of national cancer control plans, and in the effective implementation of cancer screening programmes for breast, cervical and colorectal cancers, as well variations in diagnostic practice (e.g. with respect to prostate and thyroid cancer detection) [33,34]. National strategies need to be applied or further implemented to reduce the extent of the cancer burden in Europe.

The cancer-data collection process incurs inevitable delays of several years in order to ensure the harmonisation, completeness, and quality of data necessary for the effective monitoring and control of cancer. Estimates are therefore an efficient interim means of providing a timely snapshot of the cancer situation prior to the availability of up-to-date data. The methodology used in this article is consistent with that previously applied [[9], [10], [11], [12]] and utilises the most recently collected data, such as national mortality rates for the years 2004–2018, and national and regional incidence rates for the period 2003–2018. Such a short horizon of prediction increases the accuracy and reliability of prediction provided that the baseline data is sufficient in terms of availability and accuracy.

Owing mainly to the difficulties in ascertaining and certifying the cause of death and incompleteness of death registrations, cancer mortality data are generally associated with a lower accuracy (and thus validity) than cancer-registry statistics. This may have led to under- or overestimation of the true mortality of cancer for specific sites and to the subsequent bias in incidence estimates when applying mortality to incidence ratios to obtain 2020 incidence. Sensitivity analyses were applied in such cases: incidence estimates obtained based on local incidence data were compared with incidence estimated using mortality to incidence ratios. Results obtained using the former approach were then chosen for selected cancer sites if necessary.

As performed previously for the 2018 European estimates [12], we also implemented the redistribution of ill-defined causes of deaths and of ill-defined cancers (other than the usual category "uterus unspecified") across specific cancer sites, which may have led to overestimated mortality. While compatibility with previous 2018 estimates [12] is granted, direct comparison with other estimates is not straightforward, although another set of 2020 estimates for cancer mortality in the EU-27 plus United Kingdom was published in 2020 [35] reporting an estimated total number of cancer deaths of 1.43 million (in line with 1.44 million cancer deaths reported in the present study). These figures are very similar despite the different approaches and the redistribution of the ill-defined causes of death and of ill-defined cancers across specific categories (especially on uterine cancer estimates) applied in our analysis. When considering site-specific results, the inclusion of metastatic cancers along with primary neoplasms may have overestimated mortality rates for some sites, including liver, lung and brain.

Screening programmes and specific diagnostic practices in place in Europe may have inflated regional and national differences in incidence rates observed especially in some cancers: the interpretation of breast, prostate and thyroid cancer variations should be treated with particular care. Similarly for bladder cancer, which may include some carcinomas in situ or tumours of uncertain or unknown in incidence (but not in mortality) depending on the definition of malignancy in each cancer registry [12]. In addition, several sources of data were used in generating the incidence statistics. Incidence rates at national level were provided by 25 European countries, representing 50% of the total European population.

If the size of the population covered by local registries was sufficiently large (≥60% of the national population), the estimated incidence was predicted using local incidence data (method 1, as for example the case for Germany). This was considered preferable to the calculation making use of mortality-to-incidence ratios (methods 3A and 3B, as in the case of other 15 countries) since the latter requires the additional constraint of stability in the mortality to incidence ratios on the most recent historical data. Moreover, estimation method 3A, in which all incidence and mortality data come from the same country, should be considered more reliable than method 3B, where the mortality to incidence ratio comes from neighbouring countries.

Despite the possible limitations of the data and methodology described above, the estimates provide a generally balanced overview of the European cancer burden and the associated geographical variations, and they are a valuable and appropriate means of identifying and monitoring cancer-control actions in Europe.

It has been estimated that almost 40% of cancer cases are preventable [36] and thus cancer prevention knowledge and action is crucial (https://ec.europa.eu/jrc/en/health-knowledge-gateway). The European Code Against Cancer [37] is a flagship initiative promoting preventive measures in fighting cancer. The Code describes twelve preventive actions that could avoid almost half of all European cancer deaths if respected. The first recommendation concerns smoking habits and tobacco control and is arguably the most important recommendation considering that lung cancer is the leading cause of cancer death in Europe, and furthermore that geographic variations in rates are largely determined by past exposure to tobacco smoking. Among males, incidence and mortality rates of lung cancer show decreasing trends in many European countries, especially in Northern and Western Europe, while in Central and Eastern Europe rates are still high but tending to stabilise or decline [38]. In contrast, female lung cancer incidence rates are still rising in Europe (e.g. France, Spain), although starting to stabilise, notably in the high-risk Nordic countries [38]. The more recent history of tobacco exposure among women can explain the different geographical patterns and time trends compared with males.

Incidence and mortality of tobacco-related cancers (mainly lung cancer, but also cancers of the head and neck, oesophagus, pancreas and urinary tract) could largely be avoided by primary prevention interventions on smoking habits and tobacco use [39,40]. At the European level, policies on tobacco control are led by the WHO Framework Convention on Tobacco Control (WHO FCTC) [41] and the European Tobacco Products Directive (TPD) [42], which facilitates EU Member States to transpose the directives into national law. Among the 50 countries in the WHO European Region having ratified the WHO FCTC, only half of them have raised excise taxes on tobacco products. Moreover, only 14 countries have introduced laws on smoke-free public places, and only eight countries offer cessation programmes [43].

The 2020 estimates place colorectal cancer as the second leading cause of cancer death in males, and the third in females. Risk for colorectal cancer has been associated with diets high in red meat and processed meat, overweight, smoking, and alcohol consumption [44]. Persons with a family history of colorectal cancer are also at higher risk [45]. Protective factors are a diet rich in whole grains, fruits and vegetables, as well as maintenance of a healthy life style, abstinence from smoking, limited alcohol intake and regular physical activity [40]. An IARC Handbook Working Group reported there was sufficient evidence that screening for colorectal cancer with currently established stool-based tests (guaiac testing and faecal immunochemical test [FIT]) and lower endoscopy (sigmoidoscopy and colonoscopy) reduce the risk of death from colorectal cancer and that the benefits outweigh the harms associated with each type of screening [46].

Breast cancer is the most diagnosed cancer in the female population across all European countries, and is the first cause of cancer death in women in Europe for 2020. However, a number of factors contribute to the observed geographical variations of breast cancer incidence, including implementation of organised and opportunistic screening activities, different prevalence and distribution of the major risk factors (e.g. parity, maternal age at first birth [47]), and possible methodological artefacts applied to countries with little or no data. Breast-cancer mortality rates are nevertheless decreasing in most European countries, especially in Northern and Western Europe [48]. The favourable mortality trends reflect the advances in earlier detection (due both to screening and increasing breast cancer awareness), improvements in treatment [49,50], and the possible declining use of hormone replacement therapy [HRT] after 2003 [35].

Prostate cancer is the fourth most common cancer diagnosed in Europe for 2020. Prostate cancer diagnoses followed the rapid increase in the detection of early-stage prostate cancers during the early to mid-1990s with the introduction of PSA testing, which is largely responsible for the increase of prostate cancer incidence levels [50,51]. After increasing for many years, prostate cancer incidence trends are stabilising or decreasing in the last observed 5 years (approximately after 2005), likely due in part to clinical caution in use of PSA testing given intrusive investigation and the possibility of unnecessary diagnosis of clinically irrelevant disease [52]. Decreasing mortality trends of prostate cancer in several European countries can be attributed to improvements in prostate cancer treatment [35,52], while geographical differences of mortality levels are still difficult to explain and can only partially be explained by delayed and limited access to modern effective treatments [35].

Pancreatic cancer is the fourth cause of cancer death in Europe and in the EU-27 for both sexes. Considering a low and unchanging pancreatic cancer survival [23] and the stable or increasing mortality trends [53], a growing number of deaths is expected due to the ageing of the European population. The most important recognised risk factors are tobacco smoking, obesity, dietary habits, alcohol consumption and diabetes mellitus [53,54]; the different smoking prevalence patterns by generation could partly explain the increasing mortality rates among females [53].

The incidence and mortality historical data used as baseline in our prediction process refer to the period prior to the coronavirus disease 2019 (COVID-19) pandemic and therefore the applied methodology does not account for any impact of COVID-19. A future comparison of the 2020 estimates with the recorded cancer incidence and mortality data for the same year, once available, will allow quantification of possible discrepancies for which there is already some evidence. A study published in mid 2020 from the Netherlands showed a notable decrease in cancer diagnoses in 2020 compared with the preceding period [55]; this effect was observed across all age groups and almost all cancer sites, while it was most pronounced for skin cancers. At the end of the year, the Netherlands Cancer Registry estimated that the decrease of cancer incidence in 2020 was only 3.5% (https://iknl.nl/persberichten/aantal-nieuwe-kankerpatienten-in-2020-gedaald-door). A Slovenian study [56] reported a significant decrease in numbers of first referrals, which may account for the drop of 30% in cancer notifications in April 2020 during the first surge of severe acute respiratory syndrome coronavirus 2 (SARS-CoV-2) case (indicating delayed cancer diagnosis for some patients). A Danish study found a substantial reduction amounting to one-third of the number of incident cancer diagnoses during the first three months of the pandemic compared with the previous years [57]. In Northern Ireland, the number of new patients presenting a first pathological sample indicating cancer in the period March 2020–March 2021 was 14% lower than the average reported in previous years (https://www.qub.ac.uk/research-centres/nicr); in this same country the pathologic diagnoses of Barrett's oesophagus fell by 59% compared to historical rates during the first six months of the COVID-19 pandemic [58]. A JRC survey among 40 cancer registries from 22 European countries reported that cancer-screening programmes were mostly stopped or slowed down in the majority of cancer registries' areas, and that cancer diagnostic and treatment activities were severely disrupted [59]. The impact of COVID-19 on cancer mortality data is not always reported for the year 2020 and will be more difficult to ascertain due to the trade-off of possible opposing effects. On the one hand, any delay in cancer diagnosis and/or treatment will generally increase the observed number of cancer deaths, whilst on the other, a harvesting effect on late-stage cancer patients will tend to decrease cancer mortality [53].

Population-based cancer registries are the baseline source for cancer monitoring and planning of cancer control activities at population level [60]. The results presented in this study would have not been possible without the longstanding existence of more than 150 subnational and national cancer registries and their close and continued collaboration in maintaining and providing data under the coordination of the ENCR at the European level. The need for accurate, timely cancer data will only increase, yet the contemporary financial support and advocacy to registries in some countries in Europe does not mirror the increasing demand. The recent requirement to comply with the EU General Data Protection Regulation (GDPR) places additional strain on the registry's activities. This unfavourable condition requires swift and appropriate action from political decision-makers.

## Funding

No funding declared.

## Authors contribution

**Tadeusz Dyba:** Conceptualisation, Methodology, Software, Formal analysis, Writing – Original draft preparation and Review; **Giorgia Randi:** Conceptualisation, Methodology, Software, Formal analysis, Writing – Original draft preparation and Review, Visualization; **Freddie Bray:** Conceptualization, Methodology, Writing – Review; **Carmen Martos:** Data Curation, Writing – Review; **Francesco Giusti:** Data Curation, Writing – Review; **Nicholas Nicholson:** Writing – Original draft preparation and Review; **Anna Gavin:** Writing – Review; **Manuela Flego:** Writing – Review; **Luciana Neamtiu:** Writing – Review; **Nadya Dimitrova:** Writing – Review; **Raquel** Negrão Carvalho**:** Conceptualisation, Writing – Review; **Jacques Ferlay:** Conceptualisation, Methodology, Software, Writing – Review, Supervision; **Manola Bettio:** Conceptualization, Methodology, Writing – Original draft preparation and Review, Supervision.

## Conflict of interest statement

None declared.

## Figures and Tables

**Appendix Table A1a:** Estimated number of new cancer cases (hundreds) and ASRs^a^ (per 100,000) of cancer incidence by sex, cancer site and country in Europe for 2020.

Country/region	Lip, oral cavity and pharynx				Oesophagus				Stomach				Colorectum				Liver			
	C00–14				C15				C16				C18–21				C22			
	M		F		M		F		M		F		M		F		M		F	
	Cases	ASR	Cases	ASR	Cases	ASR	Cases	ASR	Cases	ASR	Cases	ASR	Cases	ASR	Cases	ASR	Cases	ASR	Cases	ASR
**Europe**	**933.4**	**27.2**	**352.9**	**8.4**	**404.2**	**12.3**	**125.8**	**2.9**	**837.2**	**26.2**	**523.2**	**12.1**	**2817.1**	**89.0**	**2381.1**	**55.4**	**580.8**	**18.0**	**295.5**	**6.8**
**EU-27**	**560.4**	**25.6**	**238.6**	**9.3**	**232.9**	**10.9**	**70.4**	**2.7**	**466.6**	**22.4**	**287.8**	**10.6**	**1910.5**	**91.6**	**1503.7**	**56.3**	**419.1**	**19.8**	**190.2**	**7.0**
**Central and Eastern Europe**	**409.0**	**34.2**	**108.6**	**6.7**	**125.2**	**11.0**	**29.2**	**1.8**	**399.3**	**38.5**	**255.9**	**15.7**	**891.9**	**88.1**	**837.6**	**51.4**	**151.8**	**14.6**	**96.0**	**5.9**
Belarus	15.3	39.7	2.2	4.3	5.0	13.5	0.5	1.0	16.4	51.9	11.0	21.0	27.2	89.5	28.0	53.4	3.7	11.0	1.7	3.3
Bulgaria	6.7	19.5	2.3	5.4	1.8	5.2	0.5	1.1	8.1	25.0	4.6	10.5	27.0	83.8	19.5	44.9	4.2	12.5	2.2	5.2
Czechia	12.0	24.1	5.6	9.6	5.7	11.6	1.2	2.1	7.8	17.5	5.8	9.8	50.2	110.6	35.1	58.8	6.6	14.5	3.6	6.0
Hungary	17.7	39.5	6.9	12.1	5.0	11.6	1.2	2.0	10.4	26.6	7.4	12.5	55.0	135.6	42.9	73.0	7.2	17.7	3.2	5.5
Republic of Moldova	5.8	39.0	0.6	3.7	1.3	9.3	0.1	0.7	4.3	34.0	2.3	12.5	12.0	93.7	7.9	43.8	4.1	30.0	1.7	9.8
Poland	57.3	34.4	21.6	10.3	14.4	8.9	4.0	1.9	40.2	27.2	23.7	11.3	148.4	99.9	107.6	51.2	16.7	11.2	11.7	5.5
Romania	37.6	41.0	7.3	6.8	6.6	7.4	1.6	1.5	27.0	33.1	12.7	11.6	76.1	93.1	53.3	48.9	23.8	28.5	12.4	11.3
Russia	185.0	33.5	48.1	6.0	64.3	12.5	17.5	2.2	223.1	48.1	150.5	19.1	356.8	78.0	415.3	52.3	67.9	14.6	49.2	6.3
Slovakia	10.1	41.3	2.0	7.3	3.3	14.1	0.5	1.7	7.3	37.6	4.9	17.8	28.5	141.3	19.7	71.1	4.4	23.3	1.9	7.0
Ukraine	61.6	34.9	12.1	4.8	17.7	10.3	2.2	0.9	54.7	35.0	33.1	13.1	110.7	72.9	108.3	42.5	13.3	8.3	8.3	3.3
**Northern Europe**	**109.8**	**22.4**	**56.4**	**10.3**	**90.7**	**19.1**	**39.0**	**6.8**	**72.6**	**15.5**	**42.1**	**7.4**	**444.6**	**94.2**	**371.7**	**66.1**	**74.5**	**15.7**	**44.7**	**7.9**
Denmark	7.8	27.4	3.6	12.0	3.8	13.6	1.5	4.8	3.7	13.9	1.9	6.3	31.6	117.8	26.1	83.9	4.1	14.8	2.4	7.6
Estonia	1.3	24.2	0.4	5.3	0.7	13.2	0.2	1.7	2.1	42.2	1.7	19.5	4.5	94.8	5.1	58.8	0.9	17.3	0.5	6.2
Finland	4.5	16.6	2.8	8.4	2.6	9.3	1.0	2.8	3.2	12.3	2.4	7.0	19.8	74.6	18.0	53.5	4.2	15.7	2.1	6.0
Iceland	0.2	11.3	0.1	4.9	0.2	15.7	0.1	4.1	0.2	12.3	0.1	8.2	1.1	77.8	0.9	61.4	0.2	12.1	0.1	5.4
Ireland	3.7	19.4	1.7	8.7	3.6	21.2	1.7	9.0	4.6	27.1	2.3	11.9	18.6	109.6	13.2	68.1	3.1	18.5	1.6	8.6
Latvia	3.0	37.8	0.7	5.6	1.2	15.5	0.2	1.9	3.1	42.9	2.2	17.4	8.5	124.1	8.9	69.6	0.9	11.9	0.6	4.8
Lithuania	3.3	27.9	0.9	4.8	1.9	16.3	0.4	2.1	4.8	44.7	3.9	21.0	9.3	89.4	9.6	52.0	1.7	15.8	1.1	5.7
Norway	4.5	18.7	2.3	9.0	2.6	11.0	0.8	3.2	3.0	13.4	1.7	6.3	25.5	112.5	24.2	92.7	2.8	12.3	1.3	4.9
Sweden	8.1	16.7	5.2	9.8	4.1	8.6	1.8	3.1	4.9	10.2	3.0	5.4	35.5	73.7	32.3	58.8	6.5	13.2	3.3	5.9
United Kingdom	73.3	23.4	38.6	11.2	69.8	23.0	31.3	8.7	42.8	14.3	22.9	6.4	288.9	95.3	232.3	65.9	50.0	16.4	31.6	8.9
**Southern Europe**	**158.2**	**20.5**	**66.9**	**7.1**	**48.9**	**6.4**	**14.2**	**1.5**	**186.3**	**24.9**	**119.3**	**12.2**	**710.1**	**94.2**	**525.8**	**55.3**	**169.4**	**22.2**	**78.6**	**8.1**
Albania	1.3	11.7	0.8	7.4	0.5	4.4	0.1	0.8	3.4	29.6	1.3	10.8	2.1	18.4	1.8	14.4	1.0	8.9	0.7	5.6
Bosnia and Herzegovina	2.6	17.2	1.2	6.9	1.0	6.6	0.2	1.3	4.8	33.9	2.8	15.8	10.9	75.2	7.8	43.8	3.0	21.5	2.5	14.1
Croatia	4.6	22.6	1.4	5.7	1.7	8.8	0.4	1.6	5.3	28.9	3.7	14.4	22.3	119.6	14.8	57.7	4.0	21.2	1.9	7.1
Cyprus	0.4	9.0	0.2	3.7	0.2	3.4	0.0	0.8	0.7	15.9	0.5	10.5	3.7	88.1	1.7	34.0	0.7	16.7	0.4	7.9
Greece	7.2	13.4	3.3	4.7	1.8	3.4	0.6	0.9	11.3	20.9	6.5	9.3	45.3	83.6	33.7	49.1	12.0	22.2	6.3	9.1
Italy	52.5	16.6	27.2	7.1	15.1	4.8	6.5	1.6	85.5	27.4	58.2	13.9	255.9	81.6	229.9	57.4	78.6	24.9	38.8	9.4
North Macedonia	0.7	10.3	0.3	3.0	0.4	4.7	0.1	0.6	2.2	28.9	1.1	12.2	4.6	58.0	4.9	51.1	1.3	16.3	0.7	7.5
Malta	0.3	12.7	0.1	5.4	0.2	9.5	0.1	1.8	0.5	23.8	0.2	9.6	1.7	81.0	1.2	49.5	0.3	12.3	0.1	4.6
Montenegro	0.6	23.1	0.3	10.7	0.2	8.8	0.1	3.2	0.6	22.9	0.2	6.3	1.9	81.3	1.4	46.8	0.3	14.0	0.2	8.0
Portugal	17.3	34.0	4.0	6.1	6.0	11.9	0.9	1.3	17.4	35.6	12.1	17.9	64.2	130.9	40.8	61.5	11.4	23.1	4.1	5.9
Serbia	11.0	28.7	3.4	7.4	2.7	6.7	0.8	1.6	8.3	23.1	4.5	9.6	37.9	99.5	22.0	47.1	5.6	15.0	4.4	9.4
Slovenia	3.0	28.4	0.8	6.4	0.8	8.0	0.2	1.7	2.8	29.0	1.7	13.3	13.0	133.3	7.2	58.7	2.4	24.1	1.0	7.8
Spain	56.6	25.3	23.9	8.8	18.4	8.3	4.2	1.6	43.3	20.2	26.5	9.5	246.1	114.4	158.3	58.4	48.8	22.1	17.7	6.4
**Western Europe**	**256.5**	**26.2**	**121.0**	**10.9**	**139.4**	**14.5**	**43.5**	**3.8**	**179.0**	**18.9**	**105.9**	**8.9**	**770.5**	**81.5**	**645.9**	**54.7**	**185.1**	**19.4**	**76.2**	**6.3**
Austria	8.3	19.4	3.7	7.5	3.5	8.3	1.2	2.4	7.6	18.9	4.9	9.6	26.2	65.4	18.8	37.2	7.7	18.9	3.4	6.7
Belgium	15.3	27.6	6.1	9.9	8.1	15.2	3.2	5.1	10.3	20.0	5.6	8.6	54.4	105.1	41.5	64.5	9.1	17.0	3.8	5.8
France	113.2	36.2	48.5	13.3	41.0	13.4	12.1	3.2	47.1	15.5	24.3	6.0	257.8	85.3	222.8	56.4	83.0	27.1	32.1	8.0
Germany	92.9	20.9	48.6	9.8	60.4	13.7	18.3	3.6	93.7	21.5	59.6	11.2	308.7	70.8	266.5	50.0	66.8	15.2	28.7	5.3
Luxembourg	0.6	23.4	0.1	5.0	0.3	13.2	0.1	3.3	0.5	20.7	0.2	8.2	1.8	78.4	1.5	54.2	0.5	20.5	0.2	7.8
Netherlands	15.3	18.0	9.8	10.6	20.8	25.4	6.9	7.4	13.5	16.9	7.5	8.1	96.5	120.1	73.6	78.9	9.8	12.3	5.1	5.5
Switzerland	10.8	25.8	4.2	9.0	5.3	13.2	1.6	3.4	6.3	15.8	3.8	7.9	24.8	63.2	20.9	44.4	8.2	21.0	2.9	6.1

Country/region	Gallbladder				Pancreas				Larynx				Lung				Melanoma				Breast		Cervix uteri	
	C23				C25				C32				C33–34				C43				C50		C53	
	M		F		M		F		M		F		M		F		M		F		F		F	
	Cases	ASR	Cases	ASR	Cases	ASR	Cases	ASR	Cases	ASR	Cases	ASR	Cases	ASR	Cases	ASR	Cases	ASR	Cases	ASR	Cases	ASR	Cases	ASR
**Europe**	**38.8**	**1.3**	**86.9**	**2.0**	**702.1**	**22.2**	**699.1**	**16.0**	**349.5**	**10.3**	**49.1**	**1.2**	**3150.5**	**97.6**	**1624.8**	**38.3**	**763.1**	**23.2**	**743.2**	**18.2**	**5310.9**	**128.6**	**581.7**	**14.6**
**EU-27**	**26.6**	**1.3**	**51.0**	**1.9**	**471.3**	**22.6**	**477.6**	**17.5**	**211.8**	**9.8**	**34.5**	**1.4**	**2052.5**	**97.2**	**1130.7**	**43.9**	**554.0**	**26.0**	**509.7**	**20.8**	**3554.6**	**142.8**	**304.5**	**12.8**
**Central and Eastern Europe**	**14.0**	**1.6**	**38.6**	**2.4**	**225.8**	**21.6**	**218.0**	**13.4**	**165.8**	**14.1**	**15.5**	**0.9**	**1119.9**	**103.8**	**396.5**	**24.2**	**121.9**	**11.1**	**158.0**	**9.8**	**1587.1**	**97.6**	**323.5**	**20.2**
Belarus	0.3	1.0	1.3	2.5	6.8	20.9	5.2	9.9	6.3	16.4	0.2	0.4	36.4	107.9	5.2	10.0	3.8	10.8	5.2	9.8	47.5	89.4	8.4	16
Bulgaria	0.4	1.4	0.8	1.9	7.6	23.0	6.0	13.7	5.0	14.5	0.4	1.0	33.6	97.9	9.4	22.6	3.0	9.1	3.3	8.1	40.6	100.0	10.1	26
Czechia	1.3	3.0	2.7	4.5	12.6	27.5	12.1	20.1	4.3	8.5	0.7	1.2	41.5	88.9	24.1	40.1	13.7	29.2	12.2	21.2	77.2	132.0	7.7	13.5
Hungary	0.7	1.8	1.7	2.9	12.0	29.7	13.0	21.9	6.6	15.0	1.6	2.9	58.1	138.3	44.6	76.6	7.7	18.0	7.1	12.6	75.7	134.1	12.5	23.3
Republic of Moldova	0.1	0.5	0.2	1.2	3.0	23.6	2.7	15.4	3.3	20.7	0.2	1.0	12.8	92.2	3.0	16.7	1.0	7.3	0.8	4.7	14.9	75.7	4.8	22
Poland	3.2	2.3	7.6	3.6	29.7	19.7	29.6	14.1	27.1	16.5	4.2	2.0	182.8	120.0	112.3	53.1	17.2	10.8	20.2	9.7	246.4	119.1	38.6	18.9
Romania	1.2	1.5	1.2	1.1	17.2	20.7	15.1	13.8	18.0	19.8	1.2	1.1	90.3	105.3	30.9	28.5	7.9	8.9	7.6	7.1	120.9	113.1	33.8	32.3
Russia	4.9	1.2	16.8	2.1	102.6	21.4	105.5	13.4	67.1	12.3	5.4	0.7	509.9	102.4	128.9	16.1	48.1	9.5	74.2	9.3	750.5	93.1	153.1	19.1
Slovakia	0.8	4.2	2.3	8.6	5.5	26.8	5.3	19.3	3.8	15.7	0.2	0.8	25.3	120.0	7.9	28.1	4.2	19.4	4.0	14.2	30.8	108.6	7.0	24.2
Ukraine	1.2	0.9	4.0	1.6	28.8	18.3	23.6	9.3	24.4	13.9	1.3	0.5	129.2	80.7	30.0	11.8	15.3	9.1	23.5	9.4	182.6	72.1	47.6	19.2
**Northern Europe**	**5.9**	**1.3**	**12.8**	**2.2**	**97.1**	**20.7**	**97.4**	**17.1**	**31.6**	**6.5**	**6.7**	**1.2**	**394.1**	**83.7**	**356.4**	**63.6**	**169.4**	**35.2**	**166.1**	**30.6**	**831.8**	**153.7**	**66.7**	**12.6**
Denmark	0.2	0.9	0.4	1.1	5.8	21.5	6.0	19.2	2.0	7.1	0.5	1.5	24.0	88.3	26.5	85.1	13.8	50.7	15.0	51.3	50.8	171.2	3.8	13.4
Estonia	0.1	1.5	0.2	1.7	1.4	27.7	1.6	18.0	0.6	10.9	0.1	0.9	5.8	117.3	2.3	27.7	1.2	24.2	1.5	20.2	8.4	109.6	2.0	27.4
Finland	0.6	2.0	0.8	2.4	6.9	25.7	7.2	20.8	1.0	3.7	0.2	0.5	18.2	67.1	11.1	32.8	10.9	40.8	10.0	32.8	52.3	168.5	1.9	6.67
Iceland	0.0	0.9	0.0	1.6	0.3	21.2	0.2	16.8	0.1	6.0	0.0	0.7	1.0	72.1	1.1	74.3	0.2	16.1	0.3	16.0	2.3	151.1	0.2	9.92
Ireland	0.2	1.3	0.4	2.3	3.6	22.2	3.1	16.5	1.8	10.1	0.3	1.3	16.4	98.6	16.3	85.1	5.4	30.3	7.7	37.6	34.3	164.9	3.4	15.6
Latvia	0.1	0.7	0.1	1.0	1.9	26.4	2.2	16.4	1.1	13.9	0.1	0.7	8.9	127.9	3.2	24.7	1.1	15.2	1.2	10.3	12.6	108.9	2.7	25.1
Lithuania	0.1	1.3	0.4	2.3	2.6	24.3	3.2	17.1	1.5	12.9	0.1	0.7	11.1	103.2	3.9	21.3	2.1	18.7	3.1	18.2	17.7	106.5	4.1	26.8
Norway	0.2	0.9	0.3	1.1	4.7	20.8	4.5	17.4	1.0	4.1	0.2	0.9	16.6	73.8	16.5	63.9	13.0	55.4	12.7	48.6	36.7	141.8	4.0	14.9
Sweden	0.9	2.0	1.2	2.2	10.2	21.2	11.2	20.0	1.3	2.7	0.2	0.4	21.6	44.8	24.1	43.9	21.5	44.6	21.2	41.3	75.3	147.7	6.6	13.3
United Kingdom	3.5	1.2	8.9	2.5	59.5	19.7	57.7	16.2	21.2	6.8	5.0	1.5	269.4	89.3	250.4	71.4	99.6	32.4	93.0	27.1	538.9	157.5	37.9	11.2
**Southern Europe**	**10.2**	**1.4**	**17.7**	**1.8**	**152.5**	**20.3**	**155.9**	**15.9**	**81.7**	**10.6**	**11.0**	**1.2**	**740.1**	**98.0**	**303.8**	**33.2**	**124.4**	**16.2**	**114.8**	**13.4**	**1201.9**	**135.7**	**90.5**	**10.8**
Albania	0.1	0.7	0.1	0.4	1.8	16.1	0.9	7.6	1.3	10.5	0.2	2.0	9.2	82.0	1.9	15.6	0.4	2.8	0.2	1.6	9.4	71.0	1.3	10
Bosnia and Herzegovina	0.6	4.9	1.1	6.3	2.8	19.0	2.4	13.3	2.4	14.9	0.4	2.2	19.8	131.3	5.3	29.4	1.1	7.7	1.4	8.1	15.5	86.3	3.1	18
Croatia	0.6	3.2	1.1	4.1	4.3	23.0	4.4	16.8	2.7	13.1	0.2	1.0	22.8	118.4	9.6	38.5	3.8	19.6	3.2	13.5	28.9	120.3	3.4	14.7
Cyprus	0.1	2.0	0.1	2.3	1.0	23.4	0.7	14.8	0.5	9.9	0.0	0.8	4.5	102.1	1.3	24.6	0.5	10.0	0.4	7.4	7.6	140.4	0.5	7.9
Greece	0.4	0.7	0.5	0.6	12.4	22.9	10.6	15.6	7.4	13.7	0.8	1.1	67.9	127.2	21.7	33.9	7.5	13.8	5.7	9.3	77.7	124.4	7.0	11.6
Italy	4.0	1.3	6.7	1.6	64.3	20.6	77.3	18.3	27.7	8.8	4.6	1.2	283.7	90.9	135.8	35.1	68.5	21.8	56.6	16.5	551.3	151.1	31.5	9.36
North Macedonia	0.1	1.2	0.3	3.4	1.7	22.9	1.1	11.9	1.7	18.8	0.2	1.5	8.9	103.3	2.4	25.4	1.2	12.6	0.8	8.8	9.9	99.2	1.1	10.9
Malta	0.0	0.6	0.0	0.7	0.5	25.5	0.5	18.2	0.2	10.3	0.0	1.6	2.0	100.3	0.6	23.9	0.3	12.6	0.3	11.1	4.0	171.3	0.1	5.65
Montenegro	0.0	1.8	0.1	2.3	0.6	27.7	0.4	14.2	0.6	24.2	0.1	3.3	3.2	123.8	1.3	40.4	0.3	11.1	0.3	8.8	3.8	123.1	1.1	36.1
Portugal	0.5	1.0	1.0	1.4	9.4	19.3	8.5	12.2	4.8	9.6	0.5	0.7	39.3	79.5	14.8	22.8	5.1	10.2	5.7	9.4	70.4	116.9	8.7	15.1
Serbia	0.9	2.5	2.0	4.1	8.5	22.8	7.4	16.0	6.6	16.6	0.9	1.8	54.1	136.4	26.4	55.2	6.0	15.1	5.1	11.2	67.2	145.3	12.1	26.5
Slovenia	0.2	2.6	0.5	4.0	2.4	24.5	2.2	17.7	0.9	8.5	0.1	1.0	9.3	92.9	5.5	45.3	3.8	37.5	3.6	31.2	14.1	121.2	1.0	9.42
Spain	2.8	1.3	4.5	1.5	42.7	19.8	39.4	14.2	25.0	11.3	3.1	1.2	214.8	99.1	77.1	29.9	25.8	11.6	31.4	12.3	340.9	132.0	19.6	7.81
**Western Europe**	**8.7**	**0.9**	**17.8**	**1.5**	**226.7**	**24.0**	**227.9**	**18.8**	**70.4**	**7.2**	**16.0**	**1.5**	**896.5**	**93.7**	**568.1**	**50.6**	**347.5**	**36.4**	**304.2**	**28.8**	**1690.2**	**156.5**	**101.0**	**9.86**
Austria	0.4	1.1	0.9	1.8	9.8	24.3	10.3	20.2	2.3	5.4	0.5	1.1	29.9	72.8	22.6	46.3	10.3	24.9	10.1	21.3	59.2	122.7	3.9	8.14
Belgium	0.4	0.8	0.9	1.4	10.6	20.5	10.7	16.2	5.2	9.5	1.0	1.7	64.3	123.5	32.2	52.5	14.2	26.3	19.5	32.6	117.3	194.0	6.4	10.8
France	2.3	0.8	4.2	1.1	71.2	23.5	73.4	18.1	27.1	8.8	5.5	1.5	319.4	103.9	163.6	44.2	86.9	28.4	77.6	22.0	580.8	164.4	33.8	9.85
Germany	4.5	1.0	9.5	1.7	108.5	24.9	107.0	19.5	27.8	6.3	6.8	1.4	384.4	87.1	263.7	52.4	172.6	39.9	142.1	31.2	697.0	145.0	46.7	10.7
Luxembourg	0.1	2.6	0.0	0.9	0.6	28.6	0.5	16.9	0.3	10.8	0.0	1.5	2.4	102.6	1.1	40.6	0.8	30.6	0.6	20.8	5.0	171.6	0.2	8.05
Netherlands	0.7	0.9	1.4	1.5	16.7	21.1	16.6	17.6	5.8	6.9	1.6	1.7	70.5	90.0	64.5	69.4	44.2	54.2	38.9	43.7	157.3	174.4	7.7	9.1
Switzerland	0.4	1.0	0.9	1.9	9.3	23.5	9.4	19.7	1.9	4.5	0.5	1.1	25.3	64.3	20.2	43.8	18.2	44.8	15.4	33.7	72.9	160.8	2.4	5.2

European areas are reported in bold.

a ASR = Age-standardised rates using European standard population 2013, based on estimated number of new cases; M = Males, F = Females.

**Appendix Table A1b:** Estimated number of new cancer cases (hundreds) and ASRs^a^ (per 100,000) of cancer incidence by sex, cancer site and country in Europe for 2020.

Country/region	Corpus uteri		Ovary		Prostate		Testis		Kidney				Bladder				Brain, CNS			
	C54		C56		C61		C62		C64–65				C67				C70–72			
	F		F		M		M		M		F		M		F		M		F	
	Cases	ASR	Cases	ASR	Cases	ASR	Cases	ASR	Cases	ASR	Cases	ASR	Cases	ASR	Cases	ASR	Cases	ASR	Cases	ASR
**Europe**	**1300.5**	**31.2**	**666.9**	**16.0**	**4733.4**	**148.1**	**250.6**	**6.7**	**858.2**	**25.9**	**527.9**	**12.5**	**1566.6**	**50.5**	**473.3**	**10.9**	**361.9**	**10.6**	**309.2**	**7.5**
**EU-27**	**733.3**	**28.9**	**394.1**	**15.5**	**3355.1**	**158.7**	**176.4**	**8.0**	**552.3**	**25.8**	**316.1**	**12.1**	**1215.5**	**58.9**	**359.4**	**13.4**	**238.5**	**11.1**	**197.2**	**7.8**
**Central and Eastern Europe**	**592.9**	**36.1**	**285.3**	**17.6**	**1116.7**	**114.4**	**66.3**	**4.6**	**293.0**	**25.6**	**204.8**	**12.5**	**387.6**	**39.4**	**122.6**	**7.5**	**119.8**	**10.0**	**114.4**	**7.1**
Belarus	21.7	40.2	9.3	17.7	40.8	135.9	1.5	3.3	12.3	32.8	6.9	12.9	10.4	35.7	2.1	4.1	2.8	6.7	3.1	6.0
Bulgaria	13.4	32.5	6.8	16.9	49.8	156.6	2.3	6.7	5.4	16.0	3.0	7.3	14.0	43.3	4.5	10.5	4.3	12.8	3.6	8.7
Czechia	21.1	35.7	9.9	16.8	91.2	188.4	4.9	8.9	21.2	44.8	12.0	20.1	22.2	49.7	7.1	11.7	5.1	10.3	4.0	6.8
Hungary	17.0	29.5	11.8	20.9	62.3	155.1	4.8	9.4	12.3	28.4	8.3	14.3	21.4	53.6	11.0	18.9	4.1	9.3	3.6	6.4
Republic of Moldova	5.2	25.3	2.4	11.6	8.1	73.8	0.3	1.3	2.3	15.2	1.3	7.1	4.3	36.0	0.9	5.5	1.6	9.6	1.5	7.1
Poland	98.7	47.8	46.7	22.6	180.8	126.0	13.1	6.5	37.3	23.7	23.7	11.3	85.3	60.3	30.0	14.1	24.1	14.6	20.0	9.7
Romania	23.6	21.9	19.1	17.9	80.6	101.9	4.2	4.1	17.2	19.5	10.3	9.5	41.4	50.8	9.9	9.1	9.9	11.0	8.5	7.9
Russia	284.4	35.1	131.9	16.4	464.5	106.2	26.3	3.8	139.6	25.9	111.3	13.8	137.0	29.9	42.6	5.4	52.7	9.3	55.9	7.0
Slovakia	10.8	37.9	6.0	21.2	25.0	133.2	2.6	9.1	8.0	36.1	5.4	19.0	8.8	46.1	3.3	12.0	3.0	13.3	2.5	9.1
Ukraine	97.1	37.9	41.4	16.5	113.6	81.1	6.3	3.0	37.4	21.6	22.6	8.9	42.9	29.1	11.2	4.4	12.2	6.3	11.8	4.8
**Northern Europe**	**178.7**	**32.7**	**94.6**	**17.2**	**934.3**	**195.2**	**40.7**	**7.8**	**134.1**	**27.9**	**82.7**	**14.9**	**171.5**	**36.9**	**61.8**	**10.8**	**54.0**	**11.0**	**41.6**	**7.6**
Denmark	9.5	30.9	4.6	15.0	47.6	172.9	3.0	10.6	7.4	26.6	3.8	12.6	17.8	66.7	5.4	17.3	2.9	10.5	2.3	7.5
Estonia	2.3	29.0	1.5	19.1	12.3	245.4	0.2	3.3	2.3	45.3	1.5	18.6	2.2	47.5	0.9	10.3	0.6	11.1	0.6	7.7
Finland	8.9	27.0	4.9	15.1	57.1	208.5	2.0	7.4	6.5	24.0	4.2	12.5	10.0	38.2	2.5	7.2	2.5	9.1	2.1	6.6
Iceland	0.4	28.4	0.2	11.5	2.2	159.3	0.1	5.1	0.4	30.5	0.2	16.2	0.7	53.8	0.2	12.2	0.2	12.7	0.1	9.5
Ireland	8.1	40.3	4.8	23.9	45.0	250.9	2.0	9.1	6.7	37.5	3.1	15.8	7.3	44.4	2.8	14.5	2.2	11.7	2.0	9.6
Latvia	3.8	32.3	3.0	26.0	15.3	219.2	0.3	3.7	2.9	37.4	2.4	19.2	3.8	54.5	1.9	14.9	1.0	13.0	1.2	10.5
Lithuania	8.0	47.1	3.5	20.9	22.4	198.0	0.4	3.2	4.7	42.6	3.4	19.6	4.4	42.9	1.6	8.4	1.9	16.4	1.8	10.7
Norway	8.6	33.2	4.3	16.7	52.3	222.4	3.4	12.0	7.4	30.7	3.3	12.7	12.7	57.4	4.5	17.4	2.7	11.0	1.8	7.0
Sweden	14.7	27.5	7.0	13.2	109.5	223.1	3.8	7.5	9.5	19.4	5.2	9.9	22.4	46.7	7.3	13.1	4.8	9.8	3.6	6.9
United Kingdom	113.9	33.2	60.6	17.4	567.8	186.1	25.4	7.6	85.9	28.0	55.2	15.7	89.7	30.0	34.6	9.6	35.0	11.2	25.9	7.5
**Southern Europe**	**240.0**	**26.8**	**127.8**	**14.3**	**982.1**	**129.8**	**50.5**	**6.8**	**179.5**	**23.4**	**93.4**	**10.2**	**488.9**	**65.3**	**125.9**	**13.2**	**85.1**	**11.1**	**70.6**	**7.9**
Albania	2.0	15.0	0.8	5.6	7.9	73.9	0.8	5.3	1.6	13.7	0.6	4.8	2.9	28.1	0.9	8.0	1.5	11.9	0.7	5.5
Bosnia and Herzegovina	3.7	20.0	3.2	17.6	8.9	72.4	0.7	4.3	2.4	15.6	1.2	6.6	4.4	31.1	1.8	10.0	2.5	16.7	2.2	12.2
Croatia	7.9	31.9	4.9	20.2	24.8	128.5	1.9	9.3	5.7	29.4	3.2	12.9	10.5	56.3	3.5	13.9	2.6	13.3	2.2	9.0
Cyprus	1.5	28.9	0.7	13.7	8.2	199.6	0.5	6.9	0.6	12.8	0.4	7.0	1.1	26.1	0.5	9.1	0.6	11.1	0.4	8.2
Greece	21.2	34.2	10.2	16.1	62.2	116.2	3.2	6.1	12.1	22.3	6.0	9.2	48.4	90.2	8.0	11.9	7.3	13.5	6.7	10.4
Italy	100.1	27.0	53.7	14.4	393.2	124.5	23.8	8.0	76.4	24.2	46.7	12.2	225.2	72.0	58.1	14.4	31.2	10.0	26.2	7.1
North Macedonia	3.7	36.4	1.1	11.3	7.9	109.3	0.7	5.8	0.6	7.6	0.3	2.9	3.0	37.2	0.7	7.7	2.1	23.6	1.0	10.0
Malta	0.8	32.0	0.4	18.5	3.4	157.6	0.1	6.2	0.4	19.9	0.3	12.6	1.1	55.8	0.3	12.3	0.2	9.8	0.2	7.2
Montenegro	0.7	22.6	0.6	18.5	1.4	62.5	0.3	7.9	0.6	21.7	0.2	5.8	1.2	47.4	0.4	13.6	0.3	9.9	0.2	6.8
Portugal	12.4	19.7	5.6	9.0	67.6	136.5	2.4	5.2	8.1	16.4	3.8	6.0	19.0	39.2	7.1	10.3	6.5	13.3	4.5	7.3
Serbia	15.4	32.4	9.7	20.8	31.4	86.6	3.9	8.5	7.5	18.9	4.0	8.5	19.1	50.5	6.4	13.6	5.5	13.8	4.7	10.2
Slovenia	4.5	38.1	1.8	15.3	18.3	182.3	1.1	10.9	3.3	32.4	1.4	11.7	4.2	43.8	1.5	11.8	1.3	12.8	1.0	8.2
Spain	66.0	25.8	35.1	13.6	346.1	162.1	10.9	4.9	60.2	26.9	25.4	9.6	148.4	70.2	36.7	13.5	23.6	10.5	20.6	8.1
**Western Europe**	**289.0**	**25.9**	**159.3**	**14.1**	**1700.3**	**177.9**	**93.1**	**9.8**	**251.6**	**26.2**	**147.1**	**12.8**	**518.5**	**55.2**	**162.9**	**13.7**	**103.1**	**10.7**	**82.7**	**7.6**
Austria	9.5	19.5	7.8	15.9	60.9	148.5	3.6	8.1	8.4	20.1	5.2	10.4	10.9	27.6	4.3	8.6	3.8	9.0	3.2	6.6
Belgium	13.6	22.0	6.6	10.8	81.6	154.2	4.1	6.9	13.4	24.9	8.7	13.9	35.5	69.5	9.8	15.1	5.9	10.7	3.3	5.4
France	109.8	29.8	53.2	14.0	660.7	214.4	27.5	8.8	96.7	31.5	50.4	13.3	133.2	44.6	31.7	7.7	39.3	12.7	31.9	8.7
Germany	123.6	24.8	71.6	14.3	679.6	156.4	45.0	11.4	105.5	24.1	67.9	13.2	259.4	59.6	92.1	17.3	41.5	9.8	35.5	7.5
Luxembourg	0.6	21.4	0.5	17.5	3.9	165.4	0.3	8.3	0.4	15.7	0.2	8.5	0.9	41.4	0.2	8.5	0.3	10.0	0.2	5.4
Netherlands	22.0	23.7	12.5	13.4	145.8	172.7	8.3	10.1	19.9	24.1	10.3	11.1	56.7	71.5	17.5	18.7	8.1	9.6	5.8	6.4
Switzerland	9.7	21.3	7.0	15.3	67.1	165.5	4.3	9.7	7.3	18.3	4.4	9.4	21.7	56.2	7.2	15.4	4.2	10.0	2.9	6.4

Country/region	Thyroid				Hodgkin lymphoma				NHL				Multiple myeloma				Leukaemia				All sites			
	C73				C81				C82–86, C96				C88+C90				C91–95				C00–97/C44			
	M		F		M		F		M		F		M		F		M		F		M		F	
	Cases	ASR	Cases	ASR	Cases	ASR	Cases	ASR	Cases	ASR	Cases	ASR	Cases	ASR	Cases	ASR	Cases	ASR	Cases	ASR	Cases	ASR	Cases	ASR
**Europe**	**193.5**	**5.5**	**678.2**	**17.1**	**110.0**	**3.1**	**88.6**	**2.3**	**673.8**	**20.7**	**556.0**	**13.1**	**278.3**	**8.9**	**230.9**	**5.4**	**558.0**	**17.4**	**442.2**	**10.5**	**21301.8**	**660.3**	**19120.8**	**456.1**
**EU-27**	**136.8**	**6.2**	**440.3**	**18.9**	**68.7**	**3.2**	**50.9**	**2.2**	**478.6**	**22.6**	**384.6**	**14.8**	**201.0**	**9.7**	**157.5**	**5.9**	**375.6**	**18.0**	**289.9**	**11.1**	**14449.5**	**685.2**	**12370.1**	**483.6**
**Central and Eastern Europe**	**53.5**	**4.2**	**249.4**	**15.6**	**31.6**	**2.3**	**31.4**	**2.0**	**136.2**	**12.0**	**129.7**	**8.0**	**52.9**	**4.9**	**60.2**	**3.7**	**152.3**	**14.0**	**139.4**	**8.6**	**6367.1**	**600.5**	**6325.8**	**389.3**
Belarus	1.5	3.4	9.8	18.9	1.1	2.4	1.2	2.3	3.9	11.2	3.7	7.1	1.6	5.2	1.8	3.3	5.4	16.5	5.1	9.8	212.6	646.8	192.7	365.7
Bulgaria	0.7	1.9	3.2	8.6	0.6	1.7	0.7	1.9	3.5	10.4	3.0	7.3	0.9	2.6	0.9	2.1	2.9	8.9	2.6	6.5	195.4	594.5	152.0	367.7
Czechia	3.3	6.5	10.6	18.8	1.4	2.6	1.3	2.3	9.6	20.7	9.0	15.2	3.1	6.6	2.6	4.4	8.6	19.0	6.2	10.6	344.0	732.8	289.7	491.5
Hungary	2.4	5.2	10.7	20.3	1.0	2.2	1.0	1.9	7.2	17.0	8.6	14.9	2.2	5.4	2.1	3.6	7.3	18.2	6.0	10.5	315.0	760.6	309.0	540.0
Republic of Moldova	0.9	4.5	2.3	10.6	0.3	1.5	0.3	1.3	1.7	10.0	1.5	7.5	0.4	2.9	0.5	2.3	1.4	9.1	1.0	5.2	72.7	542.1	59.7	310.9
Poland	7.5	4.3	32.5	15.9	3.4	1.8	2.6	1.3	24.6	15.4	18.9	9.0	10.9	7.4	11.8	5.7	23.7	15.6	21.7	10.5	989.8	654.1	974.7	467.8
Romania	2.8	2.9	14.3	13.8	1.5	1.6	1.1	1.1	9.5	10.6	9.6	8.9	4.7	5.5	4.1	3.8	11.0	13.1	8.0	7.5	518.8	615.8	434.0	404.0
Russia	23.9	4.0	120.9	15.1	16.6	2.5	16.4	2.1	56.5	10.4	57.8	7.2	22.4	4.3	28.9	3.6	68.0	13.1	68.5	8.6	2780.5	571.6	2984.9	372.8
Slovakia	1.0	3.7	3.7	12.8	0.8	3.2	0.7	2.5	3.5	17.3	3.5	12.7	1.9	9.7	2.1	7.8	4.3	20.9	3.5	12.7	159.7	773.3	135.1	482.7
Ukraine	9.7	5.0	41.3	16.9	5.0	2.4	6.2	2.7	16.2	9.1	14.0	5.6	4.8	2.8	5.4	2.1	19.7	11.7	16.7	6.7	778.7	489.3	794.0	314.7
**Northern Europe**	**22.5**	**4.5**	**71.3**	**13.4**	**18.7**	**3.6**	**14.2**	**2.6**	**135.6**	**28.4**	**109.3**	**19.6**	**53.9**	**11.4**	**40.8**	**7.3**	**96.2**	**20.1**	**68.0**	**12.1**	**3351.3**	**703.1**	**3040.7**	**550.7**
Denmark	1.8	6.6	3.9	13.6	0.9	3.2	0.6	2.2	8.5	31.0	6.1	19.8	3.0	10.9	2.2	7.1	5.4	20.1	3.5	11.5	207.3	760.9	192.7	633.9
Estonia	0.2	3.0	0.9	11.9	0.1	2.3	0.2	2.6	1.2	22.4	1.2	14.7	0.4	9.0	0.5	5.7	1.1	20.6	0.9	11.1	40.7	817.2	36.3	451.1
Finland	1.3	4.6	4.9	17.2	0.9	3.4	0.7	2.5	7.3	27.3	6.1	18.5	2.2	8.1	2.1	6.3	4.0	15.0	2.9	8.7	176.4	654.3	159.7	495.6
Iceland	0.1	6.2	0.2	11.2	0.0	1.2	0.1	3.0	0.3	21.1	0.3	17.2	0.2	14.4	0.1	9.5	0.2	15.9	0.1	7.3	8.2	601.7	7.6	511.3
Ireland	0.7	3.3	5.0	22.9	0.7	3.5	0.6	2.9	5.1	28.4	4.0	20.1	2.0	11.8	1.4	7.0	3.6	20.3	2.7	13.1	143.8	821.7	126.9	631.2
Latvia	0.3	4.4	2.8	25.0	0.3	3.5	0.2	2.1	1.0	13.6	1.6	12.8	0.6	7.6	0.6	4.8	1.3	17.2	1.6	12.6	60.9	851.7	59.7	492.0
Lithuania	0.6	4.7	3.0	19.5	0.3	2.4	0.3	2.0	2.3	21.2	2.5	14.1	0.9	7.9	1.0	5.7	2.5	23.3	2.8	15.6	84.1	769.9	81.5	470.3
Norway	1.7	6.6	3.5	13.4	1.0	3.6	0.6	2.3	5.9	25.1	4.6	17.6	3.1	13.5	2.5	9.6	4.6	19.3	3.3	12.4	176.9	760.4	149.6	574.5
Sweden	2.1	4.3	5.4	10.9	1.2	2.4	0.9	1.9	10.4	21.6	7.8	14.4	4.8	9.8	3.3	6.0	7.9	16.3	5.5	10.1	306.6	630.9	262.3	496.0
United Kingdom	13.9	4.3	41.4	12.2	13.3	4.0	9.9	2.9	93.2	30.4	74.9	21.4	36.8	12.2	27.0	7.7	65.4	21.3	44.7	12.6	2136.5	700.4	1955.8	562.4
**Southern Europe**	**56.9**	**7.3**	**167.1**	**20.7**	**26.2**	**3.5**	**20.6**	**2.7**	**156.9**	**20.6**	**130.8**	**14.4**	**66.9**	**8.9**	**53.3**	**5.6**	**121.1**	**16.2**	**94.5**	**10.3**	**4842.3**	**640.3**	**4043.8**	**445.1**
Albania	0.2	1.6	0.8	5.3	0.2	1.7	0.3	1.9	0.4	3.0	0.2	1.3	0.3	2.2	0.1	0.8	1.3	9.6	0.9	7.1	39.9	351.6	27.1	213.4
Bosnia and Herzegovina	0.3	1.4	1.1	6.4	0.5	3.2	0.5	3.2	1.2	7.9	0.9	5.2	0.5	3.2	0.5	2.5	1.8	12.8	1.3	7.5	77.7	539.4	64.8	364.2
Croatia	1.6	7.5	5.7	25.2	0.5	2.5	0.5	2.1	2.9	15.1	2.9	11.8	1.5	8.3	1.3	5.0	2.8	16.1	2.4	9.3	135.0	709.2	115.0	467.0
Cyprus	0.6	10.1	2.3	38.4	0.2	3.0	0.2	3.6	0.9	19.0	0.9	17.9	0.4	9.7	0.4	7.3	0.8	18.3	0.6	12.4	27.4	631.7	22.5	423.9
Greece	3.7	6.9	8.8	15.2	1.8	3.3	1.8	3.2	8.8	16.3	6.8	10.8	4.8	8.8	4.4	6.5	10.6	19.7	7.8	11.8	354.2	658.6	271.6	421.7
Italy	34.5	11.0	88.4	27.9	11.7	3.9	9.5	3.1	78.6	25.1	61.7	16.3	31.4	10.0	26.3	6.6	51.5	16.6	42.0	10.9	1994.7	636.3	1832.0	485.9
North Macedonia	0.1	1.2	0.5	4.4	0.3	2.9	0.2	1.6	0.5	5.0	0.4	4.2	0.2	2.6	0.1	1.2	0.9	10.6	0.6	6.6	41.0	507.2	32.9	339.7
Malta	0.1	5.8	0.4	19.7	0.1	3.0	0.1	2.1	0.5	21.3	0.5	21.3	0.2	8.2	0.1	4.6	0.2	11.7	0.2	8.0	12.8	618.7	11.1	463.7
Montenegro	0.1	4.1	0.2	6.6	0.1	3.8	0.1	3.1	0.6	21.8	0.4	13.1	0.1	5.8	0.1	2.6	0.5	21.6	0.3	11.4	14.7	601.2	13.3	440.1
Portugal	3.0	6.0	14.0	24.9	1.5	3.0	0.9	1.7	11.5	23.3	9.5	14.9	5.5	11.2	3.4	5.2	8.4	17.4	7.1	11.1	324.4	658.5	253.1	401.7
Serbia	1.3	3.0	4.0	8.7	1.3	3.1	1.1	2.3	4.7	11.8	4.7	10.0	2.3	5.7	2.5	5.2	5.9	15.7	4.6	10.1	244.4	635.3	229.1	491.6
Slovenia	0.4	3.9	1.3	11.8	0.2	2.2	0.2	2.3	3.1	32.3	3.1	25.1	1.0	9.7	0.8	6.3	1.9	20.5	1.4	11.2	77.0	777.9	58.7	492.9
Spain	11.0	4.7	39.6	16.2	7.7	3.4	5.3	2.3	43.2	19.4	38.8	14.9	18.7	8.7	13.4	4.9	34.3	16.0	25.1	9.5	1495.1	691.2	1109.5	422.4
**Western Europe**	**60.5**	**6.2**	**190.4**	**18.8**	**33.5**	**3.5**	**22.4**	**2.3**	**245.1**	**25.8**	**186.2**	**16.2**	**104.7**	**11.1**	**76.5**	**6.5**	**188.4**	**19.9**	**140.2**	**12.1**	**6741.2**	**707.3**	**5710.6**	**510.9**
Austria	4.3	9.7	11.0	23.8	0.7	1.6	0.8	1.7	7.4	18.1	6.4	12.9	3.2	7.9	2.7	5.4	7.3	18.1	5.3	10.7	232.5	567.7	210.4	430.1
Belgium	3.1	5.5	10.2	17.3	2.2	3.8	1.4	2.4	16.6	31.3	11.7	18.7	7.2	13.7	3.7	6.0	13.5	25.4	11.1	17.5	395.0	749.8	346.7	561.3
France	28.4	9.1	102.7	30.7	12.0	3.8	8.4	2.5	83.8	27.5	60.6	15.5	38.6	12.8	31.1	7.8	66.5	21.8	50.7	12.9	2331.6	761.4	1896.7	512.4
Germany	19.1	4.5	48.1	10.9	14.4	3.5	8.7	2.1	103.3	23.9	82.2	16.0	42.2	9.7	28.6	5.4	79.3	18.6	58.7	11.6	2894.0	665.4	2487.4	499.1
Luxembourg	0.2	6.4	0.6	19.2	0.1	2.3	0.1	2.5	0.6	23.9	0.4	16.1	0.3	11.5	0.2	6.5	0.5	24.0	0.2	8.3	16.0	673.1	13.6	474.4
Netherlands	2.5	3.0	6.2	7.2	2.6	3.1	2.0	2.3	24.0	29.5	17.1	18.5	8.7	10.7	6.4	6.8	14.4	17.6	9.4	10.2	617.6	759.0	528.5	577.2
Switzerland	3.0	6.9	11.5	25.9	1.6	3.7	1.0	2.2	9.4	23.6	7.6	16.3	4.5	11.5	3.8	8.1	7.0	17.7	4.7	10.1	251.8	629.7	225.3	489.5

European areas are reported in bold.

a ASR = Age-standardised rates using European standard population 2013, based on estimated number of new cases; M = Males, F = Females; CNS = central nervous system; NHL = Non-Hodgkin lymphoma.

**Appendix Table A2a:** Estimated number of deaths (hundreds) and ASRs^a^ (per 100,000) of cancer mortality by sex, cancer site and country in Europe for 2020.

Country/region	Lip, oral cavity and pharynx				Oesophagus				Stomach				Colorectum				Liver			
	C00–14				C15				C16				C18–21				C22			
	M		F		M		F		M		F		M		F		M		F	
	Cases	ASR	Cases	ASR	Cases	ASR	Cases	ASR	Cases	ASR	Cases	ASR	Cases	ASR	Cases	ASR	Cases	ASR	Cases	ASR
**Europe**	**414.2**	**12.3**	**124.6**	**2.9**	**349.1**	**10.8**	**106.0**	**2.4**	**594.6**	**18.9**	**375.4**	**8.6**	**1318.9**	**43.5**	**1129.4**	**25.5**	**513.4**	**16.3**	**270.7**	**6.2**
**EU-27**	**238.1**	**11.0**	**79.8**	**3.0**	**198.5**	**9.4**	**57.7**	**2.1**	**321.4**	**15.7**	**199.4**	**7.2**	**871.9**	**43.1**	**689.2**	**24.5**	**366.2**	**17.6**	**172.5**	**6.3**
**Central and Eastern Europe**	**214.1**	**18.0**	**48.2**	**3.0**	**114.4**	**10.1**	**26.9**	**1.7**	**304.4**	**30.3**	**195.8**	**12.1**	**483.8**	**51.9**	**450.1**	**27.8**	**139.9**	**13.6**	**90.2**	**5.6**
Belarus	9.7	24.9	1.4	2.5	4.6	12.7	0.5	0.9	12.1	39.8	7.9	15.2	13.8	50.9	14.1	26.9	3.5	10.6	1.6	3.1
Bulgaria	3.4	9.8	0.9	2.1	1.7	4.9	0.4	1.0	6.4	20.8	4.3	10.1	16.3	53.6	11.4	26.3	3.7	11.4	2.1	4.9
Czechia	5.6	11.4	2.3	3.9	4.8	10.0	1.2	2.0	5.6	12.7	3.9	6.6	20.1	46.6	14.3	23.9	5.4	12.0	3.3	5.5
Hungary	10.5	23.8	2.9	5.0	4.3	10.0	1.1	1.8	8.0	21.1	5.7	9.6	27.3	72.0	21.5	36.1	6.3	15.7	3.1	5.2
Republic of Moldova	4.1	27.4	0.3	2.0	1.2	8.6	0.1	0.6	3.6	29.4	1.8	10.3	7.2	61.8	4.7	27.6	3.9	29.2	1.7	9.8
Poland	30.2	18.5	9.2	4.4	13.7	8.6	3.6	1.7	35.6	24.8	19.9	9.4	86.4	63.3	64.4	30.5	14.2	9.7	10.3	4.8
Romania	20.6	22.7	2.9	2.7	5.7	6.5	1.5	1.3	22.1	27.5	10.4	9.5	41.1	53.2	28.0	25.6	22.0	26.5	11.8	10.8
Russia	89.4	16.2	22.5	2.8	59.1	11.6	16.2	2.0	165.4	36.8	113.4	14.5	196.2	47.1	224.6	28.7	64.5	13.9	46.7	5.9
Slovakia	6.0	24.7	0.9	3.4	2.8	12.4	0.5	1.7	3.9	21.1	3.1	11.4	14.5	82.7	11.3	42.9	3.7	19.4	1.7	6.1
Ukraine	34.6	19.3	5.0	2.0	16.4	9.5	2.0	0.8	41.7	27.5	25.4	10.1	60.9	42.6	55.8	22.0	12.7	7.9	7.9	3.1
**Northern Europe**	**39.1**	**8.1**	**16.8**	**3.0**	**77.2**	**16.4**	**32.7**	**5.6**	**48.3**	**10.5**	**29.4**	**5.1**	**178.1**	**38.9**	**159.6**	**27.3**	**64.9**	**13.9**	**40.2**	**7.0**
Denmark	2.7	9.9	1.1	3.6	3.4	12.6	1.1	3.4	2.6	9.9	1.6	5.0	10.5	40.7	9.4	29.6	4.0	14.6	2.4	7.6
Estonia	0.8	15.7	0.1	1.4	0.6	11.3	0.1	1.5	1.5	30.1	1.4	16.7	2.5	56.9	3.0	32.8	0.8	15.3	0.5	5.9
Finland	1.4	5.2	0.9	2.6	2.5	9.0	0.8	2.3	2.5	9.6	1.7	5.0	8.1	31.3	6.8	19.5	4.1	15.3	1.9	5.5
Iceland	0.0	2.9	0.0	1.4	0.2	13.6	0.1	3.2	0.1	6.5	0.1	4.6	0.4	27.9	0.3	22.5	0.2	11.6	0.1	4.9
Ireland	1.5	8.3	0.5	2.8	3.5	20.9	1.4	7.2	1.9	11.6	1.4	7.5	7.5	47.7	5.3	28.0	2.8	16.9	1.4	7.4
Latvia	1.7	21.6	0.3	2.3	1.0	13.5	0.2	1.8	2.6	38.1	1.6	12.0	3.0	45.4	3.8	28.4	0.8	10.4	0.5	3.7
Lithuania	2.7	23.0	0.5	2.8	1.7	14.9	0.3	1.8	3.7	36.3	2.5	13.3	4.5	46.1	4.5	23.7	1.6	13.9	0.9	4.8
Norway	1.1	4.8	0.5	1.9	2.1	9.4	0.7	2.6	2.0	9.3	1.0	3.6	9.3	43.4	9.3	35.1	2.7	12.0	1.1	4.3
Sweden	2.5	5.2	1.5	2.7	4.0	8.3	1.5	2.6	3.3	6.9	2.3	4.2	16.2	34.3	15.4	26.9	5.7	12.0	2.9	5.0
United Kingdom	24.6	8.0	11.3	3.2	58.0	19.2	26.5	7.3	28.0	9.4	15.8	4.3	115.7	39.1	101.2	27.6	42.2	14.1	28.4	7.9
**Southern Europe**	**65.0**	**8.5**	**24.9**	**2.5**	**43.6**	**5.7**	**12.1**	**1.2**	**131.4**	**17.7**	**83.1**	**8.3**	**315.8**	**42.8**	**238.2**	**23.5**	**144.5**	**19.2**	**68.0**	**6.8**
Albania	0.8	7.1	0.4	3.5	0.4	4.1	0.1	0.8	2.8	24.5	1.1	9.5	1.2	10.9	1.0	8.4	1.0	8.3	0.6	5.1
Bosnia and Herzegovina	1.3	8.9	0.4	2.4	0.9	6.1	0.2	1.3	3.8	28.4	2.3	13.0	6.5	49.7	4.6	26.7	2.8	21.0	2.4	13.9
Croatia	2.7	13.9	0.7	2.5	1.4	7.1	0.3	1.3	4.2	23.8	2.9	11.1	13.6	79.1	9.6	36.6	3.6	20.1	1.7	6.4
Cyprus	0.2	3.6	0.1	1.3	0.1	3.5	0.0	0.4	0.7	16.1	0.5	9.8	1.6	40.4	1.1	22.9	0.6	15.0	0.4	7.2
Greece	3.0	5.5	1.4	1.9	1.6	2.9	0.5	0.7	8.5	15.6	4.9	6.9	22.9	41.4	16.9	22.9	9.8	18.1	5.4	7.6
Italy	22.4	7.1	11.3	2.7	14.2	4.5	5.6	1.3	53.2	17.1	35.4	8.1	118.1	38.0	99.8	23.0	67.2	21.5	30.7	7.1
North Macedonia	0.4	6.0	0.1	1.2	0.3	4.2	0.1	0.6	1.9	25.3	0.9	10.6	2.5	34.8	2.5	28.4	1.2	15.2	0.6	7.3
Malta	0.1	3.9	0.1	3.1	0.2	7.5	0.0	1.5	0.3	15.6	0.2	7.1	0.7	34.4	0.6	24.0	0.2	10.4	0.1	4.0
Montenegro	0.4	15.6	0.2	7.5	0.2	7.2	0.1	2.8	0.5	22.8	0.2	5.5	1.3	62.5	0.6	21.3	0.3	12.0	0.2	7.8
Portugal	7.5	14.9	1.5	2.1	5.1	10.3	0.8	1.1	14.1	29.0	9.3	13.3	25.6	53.6	17.6	24.8	11.2	22.7	4.0	5.7
Serbia	6.0	16.1	1.4	3.0	2.6	6.5	0.7	1.5	7.0	19.9	3.7	8.1	21.1	59.6	12.4	27.0	5.4	14.4	4.2	9.1
Slovenia	1.3	12.4	0.3	2.0	0.7	7.2	0.2	1.5	2.0	21.6	1.2	9.5	4.2	47.9	3.2	24.3	2.2	22.6	0.9	7.4
Spain	18.9	8.6	7.2	2.6	15.8	7.2	3.4	1.2	32.4	15.3	20.6	7.2	96.4	46.0	68.3	23.5	38.8	18.0	16.7	5.9
**Western Europe**	**96.1**	**9.9**	**34.6**	**2.9**	**114.0**	**12.0**	**34.4**	**2.9**	**110.4**	**11.8**	**67.2**	**5.4**	**341.1**	**36.7**	**281.5**	**22.4**	**164.2**	**17.4**	**72.4**	**5.9**
Austria	4.9	11.5	1.5	2.9	3.2	7.7	1.0	2.0	4.9	12.6	3.5	6.7	13.2	34.2	9.8	18.6	7.0	17.4	3.0	5.8
Belgium	5.1	9.6	2.0	3.1	6.8	13.0	2.4	3.7	5.0	10.0	2.8	4.1	18.0	36.0	15.5	22.8	7.5	14.5	3.5	5.3
France	32.5	10.5	10.9	2.8	32.4	10.7	10.3	2.6	32.7	10.9	17.4	4.2	111.8	37.8	97.8	23.0	72.7	24.1	30.0	7.3
Germany	45.4	10.2	16.2	3.0	51.3	11.6	13.9	2.6	55.6	12.8	36.3	6.5	151.2	35.0	119.6	20.9	60.4	13.8	28.3	5.2
Luxembourg	0.1	4.2	0.0	1.2	0.2	9.8	0.1	2.2	0.3	13.3	0.2	7.0	0.7	31.9	0.6	19.0	0.4	15.6	0.2	5.5
Netherlands	4.4	5.4	2.8	3.0	15.9	19.9	5.5	5.9	7.9	10.2	4.6	4.8	36.1	47.3	29.7	31.3	9.6	12.1	4.8	5.1
Switzerland	3.7	9.0	1.3	2.7	4.1	10.5	1.2	2.5	3.9	10.1	2.4	4.9	10.1	26.7	8.6	17.3	6.6	17.2	2.5	5.4

Country/region	Gallbladder				Pancreas				Larynx				Lung				Melanoma				Breast		Cervix uteri	
	C23				C25				C32				C33–34				C43				C50		C53	
	M		F		M		F		M		F		M		F		M		F		F		F	
	Cases	ASR	Cases	ASR	Cases	ASR	Cases	ASR	Cases	ASR	Cases	ASR	Cases	ASR	Cases	ASR	Cases	ASR	Cases	ASR	Cases	ASR	Cases	ASR
**Europe**	**26.0**	**0.9**	**61.2**	**1.4**	**667.0**	**21.2**	**654.4**	**14.9**	**176.2**	**5.4**	**19.9**	**0.5**	**2600.2**	**81.7**	**1241.6**	**29.0**	**147.1**	**4.7**	**116.6**	**2.7**	**1417.7**	**32.9**	**259.9**	**6.3**
**EU-27**	**18.2**	**0.9**	**36.8**	**1.3**	**447.3**	**21.6**	**445.3**	**16.2**	**102.2**	**4.8**	**13.5**	**0.5**	**1705.6**	**81.7**	**867.3**	**33.2**	**94.6**	**4.6**	**70.3**	**2.6**	**918.3**	**34.1**	**134.4**	**5.3**
**Central and Eastern Europe**	**10.7**	**1.2**	**29.1**	**1.8**	**218.1**	**20.9**	**209.8**	**12.9**	**95.2**	**8.5**	**7.4**	**0.5**	**967.7**	**91.2**	**338.3**	**20.7**	**46.1**	**4.5**	**46.6**	**2.9**	**514.9**	**31.7**	**158.5**	**9.8**
Belarus	0.2	0.5	0.9	1.7	6.7	20.7	5.0	9.6	3.6	9.9	0.2	0.3	31.3	96.6	4.4	8.3	1.4	4.7	1.6	3.0	13.2	25.0	3.6	6.8
Bulgaria	0.3	1.0	0.5	1.1	7.0	21.7	5.4	12.5	3.3	9.7	0.3	0.6	27.8	82.5	8.3	19.7	1.3	4.1	0.9	2.1	15.3	36.3	5.0	12.4
Czechia	0.9	2.1	1.9	3.1	11.3	24.9	10.7	17.9	2.2	4.4	0.3	0.5	32.8	70.6	18.7	31.3	2.4	5.5	1.9	3.1	16.7	28.1	4.0	6.8
Hungary	0.6	1.6	1.4	2.4	11.3	28.2	12.2	20.6	3.5	8.2	0.7	1.2	52.0	125.0	37.2	63.5	1.8	4.6	1.5	2.6	22.0	37.6	4.8	8.6
Republic of Moldova	0.0	0.3	0.2	0.9	2.9	22.5	2.6	14.9	2.0	13.1	0.1	0.4	11.0	81.0	2.5	14.2	0.5	3.5	0.3	1.8	7.0	37.7	2.5	12.5
Poland	2.8	2.0	6.9	3.3	28.5	18.9	29.2	13.9	15.9	10.2	2.2	1.0	174.6	116.2	99.8	47.0	8.8	6.1	8.9	4.2	88.1	41.8	21.4	10.5
Romania	1.0	1.3	0.9	0.9	16.2	19.6	14.1	12.9	10.5	11.9	0.6	0.6	80.9	95.6	26.9	24.8	2.8	3.3	2.2	2.1	39.2	36.2	18.1	16.9
Russia	3.5	0.9	12.0	1.5	101.4	21.2	103.3	13.1	38.0	7.4	2.6	0.3	435.2	89.1	108.5	13.6	19.0	3.9	21.7	2.7	231.3	29.0	75.5	9.4
Slovakia	0.5	2.9	1.4	5.1	4.7	22.9	4.3	15.8	1.6	7.0	0.1	0.3	17.2	89.0	7.4	26.6	1.8	9.6	1.4	5.1	11.9	43.8	2.8	10.1
Ukraine	0.9	0.7	3.1	1.2	28.3	18.0	22.8	9.0	14.6	8.6	0.5	0.2	104.9	65.4	24.6	9.7	6.5	4.2	6.2	2.5	70.3	27.7	20.9	8.3
**Northern Europe**	**2.9**	**0.6**	**7.9**	**1.4**	**87.9**	**18.8**	**87.0**	**15.1**	**12.2**	**2.6**	**2.5**	**0.4**	**290.7**	**62.5**	**253.5**	**44.7**	**28.1**	**6.1**	**19.3**	**3.4**	**179.6**	**31.7**	**21.3**	**3.9**
Denmark	0.1	0.5	0.2	0.6	5.6	20.9	5.6	17.9	0.8	2.9	0.2	0.6	20.3	76.5	19.0	60.6	1.8	6.9	1.6	5.3	11.2	36.0	1.4	4.6
Estonia	0.1	1.1	0.1	1.6	1.1	22.9	1.6	17.6	0.4	7.9	0.0	0.0	4.9	100.8	1.8	20.5	0.2	4.5	0.3	3.0	2.7	31.3	0.6	7.9
Finland	0.3	1.0	0.6	1.5	6.7	25.0	7.0	19.9	0.4	1.5	0.0	0.1	14.9	55.5	9.0	26.5	1.8	6.9	0.7	1.9	8.6	26.2	0.7	2.1
Iceland	0.0	0.0	0.0	0.0	0.3	19.4	0.2	14.2	0.0	0.9	0.0	0.0	0.8	56.7	0.7	48.2	0.1	6.9	0.1	4.1	0.5	33.1	0.1	3.4
Ireland	0.1	0.9	0.3	1.6	3.6	23.0	2.6	13.7	0.7	4.4	0.1	0.5	11.9	75.2	10.4	54.6	1.0	5.8	0.9	4.4	7.5	38.1	1.1	5.1
Latvia	0.0	0.4	0.1	0.8	1.7	22.9	1.9	14.2	0.6	8.3	0.0	0.2	6.0	82.6	2.6	20.0	0.4	4.6	0.5	3.4	4.3	34.8	1.4	11.7
Lithuania	0.1	1.2	0.3	1.3	2.3	22.1	3.0	15.6	1.2	10.6	0.1	0.4	9.2	84.9	2.8	15.3	0.6	5.1	0.6	3.3	4.9	27.8	1.9	11.6
Norway	0.1	0.4	0.2	0.6	4.5	19.8	4.2	16.2	0.3	1.5	0.1	0.3	12.5	57.2	12.3	47.1	2.1	9.1	1.7	6.3	6.3	24.1	1.0	3.7
Sweden	0.4	0.8	1.0	1.8	9.9	20.7	10.5	18.4	0.5	1.1	0.1	0.2	18.2	37.9	20.3	36.4	3.3	7.0	2.3	4.2	14.7	27.0	2.0	3.7
United Kingdom	1.7	0.6	5.2	1.4	52.0	17.3	50.2	14.0	7.2	2.4	1.9	0.5	191.1	64.0	174.1	49.0	17.0	5.7	10.7	3.0	118.4	33.1	11.2	3.2
**Southern Europe**	**7.0**	**0.9**	**12.4**	**1.3**	**144.1**	**19.2**	**141.1**	**14.3**	**40.5**	**5.4**	**4.8**	**0.5**	**616.9**	**82.3**	**239.4**	**25.6**	**29.8**	**3.9**	**19.5**	**2.1**	**286.1**	**29.7**	**37.1**	**4.1**
Albania	0.0	0.4	0.0	0.4	1.7	15.2	0.9	7.4	0.8	6.6	0.1	1.3	8.1	73.4	1.5	13.1	0.1	1.1	0.1	0.7	2.6	20.6	0.7	5.6
Bosnia and Herzegovina	0.4	3.2	0.7	4.2	2.7	19.8	2.4	13.6	1.5	10.4	0.2	1.2	17.7	120.9	4.7	26.2	0.5	3.5	0.4	2.5	5.7	32.6	1.5	8.6
Croatia	0.5	2.8	1.0	3.8	3.8	21.0	4.0	15.4	1.5	7.9	0.1	0.5	21.2	112.5	8.6	34.2	1.4	7.7	1.0	3.8	8.3	32.8	1.5	6.1
Cyprus	0.0	0.7	0.1	1.3	0.9	22.1	0.7	13.5	0.2	4.9	0.0	0.0	3.4	80.0	1.1	21.4	0.2	5.2	0.1	1.9	2.0	39.7	0.3	6.4
Greece	0.3	0.5	0.4	0.5	11.3	20.9	9.6	14.1	3.5	6.3	0.3	0.5	59.2	110.8	17.4	26.7	1.9	3.5	1.1	1.6	23.3	33.7	2.8	4.3
Italy	2.9	0.9	5.0	1.2	62.4	20.0	66.8	15.8	12.7	4.1	1.9	0.5	227.9	73.4	108.1	26.9	14.0	4.5	8.2	2.1	126.3	30.9	10.1	2.7
North Macedonia	0.1	1.0	0.2	2.6	1.7	22.8	1.1	11.7	0.9	11.4	0.1	0.9	7.8	92.8	2.0	21.4	0.5	5.5	0.3	3.4	3.2	34.8	0.6	6.4
Malta	0.0	0.0	0.0	0.0	0.5	22.9	0.4	17.0	0.1	3.5	0.0	0.3	1.5	74.1	0.5	18.4	0.1	3.3	0.1	1.9	0.7	28.1	0.1	2.2
Montenegro	0.0	0.8	0.1	2.0	0.6	26.3	0.4	13.9	0.4	16.4	0.0	1.5	2.7	107.2	1.1	34.5	0.2	8.3	0.1	4.0	1.5	49.3	0.5	17.9
Portugal	0.4	0.8	0.7	1.0	9.3	19.0	8.4	12.1	3.1	6.2	0.2	0.3	36.2	73.6	11.8	17.8	1.5	3.2	1.4	2.0	18.6	28.0	3.8	6.0
Serbia	0.7	1.9	1.3	2.8	8.4	22.6	7.3	15.7	3.9	10.3	0.5	1.1	48.9	124.1	21.9	45.5	2.4	6.6	1.5	3.3	23.4	50.9	6.3	13.7
Slovenia	0.2	2.1	0.3	2.5	2.1	22.1	2.0	15.5	0.5	5.6	0.1	0.5	8.2	84.2	4.8	39.9	0.8	7.9	0.5	4.2	4.1	32.3	0.5	4.5
Spain	1.7	0.8	2.6	0.9	38.5	17.9	37.2	13.3	11.4	5.3	1.2	0.4	173.5	80.8	55.8	21.3	6.1	2.8	4.8	1.7	66.1	23.8	8.1	3.1
**Western Europe**	**5.5**	**0.6**	**11.8**	**1.0**	**216.8**	**23.0**	**216.5**	**17.7**	**28.2**	**3.0**	**5.2**	**0.5**	**724.9**	**76.2**	**410.4**	**35.9**	**43.0**	**4.6**	**31.2**	**2.6**	**437.1**	**36.6**	**43.0**	**3.8**
Austria	0.3	0.8	0.5	1.0	9.5	24.0	9.9	19.2	1.4	3.4	0.2	0.4	25.5	63.0	16.8	34.0	2.5	6.2	1.7	3.3	16.9	33.0	1.7	3.4
Belgium	0.2	0.3	0.5	0.7	9.7	18.9	10.6	16.0	1.6	3.0	0.3	0.5	44.4	86.2	23.9	38.1	2.2	4.1	2.0	3.1	23.6	36.1	2.4	3.8
France	0.9	0.3	1.9	0.4	67.8	22.5	70.1	17.1	9.0	3.0	1.7	0.4	252.1	82.7	118.8	31.5	12.1	4.0	9.2	2.3	141.8	35.6	14.5	3.9
Germany	3.5	0.8	7.5	1.3	105.2	24.2	101.7	18.3	13.6	3.1	2.4	0.5	316.6	72.1	186.2	36.4	18.6	4.3	13.1	2.5	205.8	38.1	20.8	4.2
Luxembourg	0.0	0.0	0.0	0.0	0.5	21.5	0.4	14.8	0.1	3.5	0.0	0.0	1.8	79.0	0.9	31.0	0.1	4.6	0.1	2.9	0.9	31.7	0.1	3.6
Netherlands	0.3	0.4	1.0	1.0	15.9	20.0	15.5	16.4	2.0	2.5	0.5	0.6	63.2	81.3	48.6	52.0	5.4	7.1	3.6	3.9	32.8	35.2	2.5	2.8
Switzerland	0.2	0.6	0.6	1.1	8.1	20.9	8.4	17.4	0.7	1.6	0.2	0.3	20.8	53.6	15.0	32.5	2.2	5.8	1.5	3.1	15.1	31.6	1.0	2.1

European areas are reported in bold.

a ASR = Age-standardised rates using European standard population 2013, based on estimated number of deaths; M = Males, F = Females.

**Appendix Table A2b:** Estimated number of deaths (hundreds) and ASRs^a^ (per 100,000) of cancer mortality by sex, cancer site and country in Europe for 2020.

Country/region	Corpus uteri		Ovary		Prostate		Testis		Kidney				Bladder				Brain, CNS			
	C54		C56		C61		C62		C64–65				C67				C70–72			
	F		F		M		M		M		F		M		F		M		F	
	Cases	ASR	Cases	ASR	Cases	ASR	Cases	ASR	Cases	ASR	Cases	ASR	Cases	ASR	Cases	ASR	Cases	ASR	Cases	ASR
**Europe**	**299.6**	**6.9**	**440.5**	**10.3**	**1080.9**	**38.2**	**15.7**	**0.4**	**346.0**	**11.1**	**194.5**	**4.4**	**508.2**	**17.5**	**164.7**	**3.6**	**293.7**	**8.8**	**243.1**	**5.8**
**EU-27**	**167.7**	**6.2**	**271.4**	**10.3**	**699.5**	**36.3**	**9.3**	**0.4**	**221.8**	**10.8**	**122.3**	**4.4**	**371.5**	**18.8**	**120.4**	**4.2**	**194.0**	**9.0**	**153.1**	**6.0**
**Central and Eastern Europe**	**136.1**	**8.3**	**175.7**	**10.8**	**348.0**	**42.9**	**8.2**	**0.6**	**127.4**	**12.5**	**75.0**	**4.6**	**148.0**	**17.3**	**41.3**	**2.6**	**98.4**	**8.5**	**93.6**	**5.8**
Belarus	4.5	8.4	5.3	9.9	10.2	44.5	0.2	0.4	4.5	14.0	2.1	4.0	3.4	13.3	0.7	1.3	2.4	5.7	2.8	5.2
Bulgaria	3.5	8.2	5.1	12.3	12.2	43.5	0.4	1.1	2.6	8.0	1.2	2.9	5.3	17.8	1.6	3.5	4.2	12.5	3.3	7.9
Czechia	4.0	6.6	6.3	10.6	14.7	38.7	0.3	0.6	7.4	16.7	4.2	6.9	6.7	16.7	2.5	4.1	3.8	7.8	3.0	5.1
Hungary	4.0	6.7	7.5	12.9	14.8	44.4	0.4	0.8	4.6	11.6	3.4	5.7	6.4	17.9	3.4	5.6	3.5	8.0	3.2	5.5
Republic of Moldova	1.4	7.3	1.5	7.7	3.7	37.9	0.1	0.4	1.2	8.7	0.6	3.1	1.9	18.5	0.4	2.6	1.3	8.2	1.2	5.9
Poland	22.0	10.6	31.3	15.0	70.7	58.8	1.5	0.8	19.9	13.9	11.4	5.3	38.6	30.6	11.6	5.4	19.4	12.1	15.6	7.5
Romania	5.2	4.7	11.2	10.4	24.4	34.9	0.6	0.6	6.7	8.1	3.9	3.6	14.0	18.7	3.7	3.4	8.9	10.1	7.7	7.1
Russia	68.9	8.6	80.4	10.0	144.3	39.8	3.4	0.6	59.7	12.8	37.1	4.7	49.1	12.5	12.9	1.7	42.5	7.8	45.0	5.6
Slovakia	2.6	9.5	3.9	14.0	10.8	75.5	0.2	1.0	3.6	19.9	2.2	8.1	5.0	32.0	1.3	5.0	2.1	9.3	2.1	7.5
Ukraine	20.2	7.9	23.1	9.1	42.2	33.3	1.2	0.6	17.3	10.8	9.0	3.5	17.6	13.3	3.4	1.3	10.4	5.5	9.8	3.9
**Northern Europe**	**41.6**	**7.3**	**65.3**	**11.6**	**209.4**	**48.2**	**1.1**	**0.2**	**47.0**	**10.1**	**28.7**	**4.9**	**66.8**	**15.0**	**28.9**	**4.9**	**43.5**	**9.0**	**32.9**	**6.0**
Denmark	2.2	6.9	3.7	11.7	13.9	58.4	0.1	0.2	2.6	9.9	1.2	3.8	4.0	15.9	1.9	6.0	2.8	10.2	2.3	7.4
Estonia	0.5	6.0	0.9	11.1	3.3	78.5	0.0	0.2	0.9	19.9	0.6	6.7	0.8	19.3	0.3	3.2	0.5	9.9	0.5	6.7
Finland	2.1	6.0	3.7	11.2	9.1	38.0	0.1	0.3	2.4	9.3	1.8	5.1	2.0	8.1	0.7	2.0	2.2	8.1	1.4	4.4
Iceland	0.1	3.5	0.2	11.2	0.6	49.8	0.0	0.0	0.1	9.6	0.1	6.1	0.2	12.2	0.0	2.6	0.1	9.4	0.1	8.9
Ireland	2.0	10.6	3.0	15.2	5.7	40.5	0.0	0.2	1.8	10.7	1.5	7.8	2.5	17.0	1.0	5.5	1.9	10.2	1.8	8.7
Latvia	1.5	11.7	1.8	14.4	4.1	65.6	0.0	0.2	1.3	18.7	1.0	7.5	2.1	33.7	0.6	4.2	0.7	9.6	0.8	6.3
Lithuania	1.6	8.4	2.6	14.6	5.6	60.2	0.1	0.5	1.8	17.2	1.2	6.1	2.0	21.0	0.6	2.8	1.2	10.8	1.4	7.9
Norway	1.9	7.1	3.0	11.7	10.6	55.3	0.1	0.3	1.8	7.9	1.2	4.4	2.6	13.2	1.0	3.9	2.2	8.9	1.3	5.1
Sweden	3.5	6.0	5.3	9.8	24.1	54.1	0.1	0.2	4.0	8.4	2.5	4.4	5.8	12.5	2.6	4.4	4.3	8.8	3.3	6.2
United Kingdom	26.1	7.3	41.0	11.6	131.7	46.3	0.6	0.2	30.1	10.1	17.5	4.8	44.6	15.4	20.0	5.4	27.4	8.9	20.0	5.7
**Southern Europe**	**56.4**	**5.8**	**80.2**	**8.6**	**203.8**	**28.2**	**2.9**	**0.4**	**67.1**	**9.0**	**34.3**	**3.4**	**140.0**	**19.2**	**39.3**	**3.8**	**68.4**	**8.9**	**54.5**	**6.0**
Albania	0.4	3.8	0.5	3.6	3.4	36.3	0.1	0.7	0.9	8.5	0.3	2.6	1.4	14.8	0.4	4.3	1.2	9.9	0.6	4.6
Bosnia and Herzegovina	1.0	5.6	2.1	11.4	4.3	38.8	0.1	0.8	1.3	9.2	0.6	3.4	2.0	16.0	0.7	3.9	2.1	14.2	1.8	10.4
Croatia	2.0	7.5	3.1	12.5	8.1	51.9	0.1	0.7	2.5	14.1	1.6	6.0	3.7	23.0	1.8	6.6	2.2	11.3	1.9	7.6
Cyprus	0.5	9.5	0.7	13.7	1.8	55.5	0.0	0.1	0.4	9.0	0.1	1.9	0.9	24.6	0.2	3.2	0.5	9.7	0.3	6.0
Greece	4.8	7.1	6.8	10.2	18.4	32.4	0.2	0.4	4.6	8.3	2.4	3.3	12.8	23.1	2.7	3.5	5.7	10.5	5.1	7.8
Italy	21.5	5.3	32.9	8.4	69.0	22.6	1.0	0.3	27.3	8.7	15.5	3.6	55.7	18.0	15.4	3.4	25.4	8.1	20.3	5.3
North Macedonia	0.8	8.6	0.7	7.2	3.0	52.3	0.1	0.5	0.3	4.5	0.1	1.6	1.0	15.5	0.3	3.3	1.7	19.5	0.8	8.9
Malta	0.2	8.6	0.3	12.2	0.5	28.6	0.0	0.0	0.2	9.1	0.1	4.0	0.3	14.4	0.1	2.8	0.2	7.2	0.1	3.6
Montenegro	0.4	12.3	0.4	12.7	0.9	50.8	0.0	0.6	0.4	16.0	0.0	1.5	0.5	26.9	0.1	3.7	0.2	8.1	0.2	5.3
Portugal	3.6	5.3	4.1	6.3	19.2	41.6	0.2	0.4	3.7	7.9	1.5	2.1	8.8	18.7	3.3	4.4	5.4	10.9	4.0	6.2
Serbia	3.8	8.2	6.3	13.3	12.5	40.1	0.5	1.1	3.8	10.0	1.8	3.9	7.0	21.0	2.3	5.1	4.6	11.7	3.9	8.4
Slovenia	0.9	6.9	1.4	11.2	4.5	56.2	0.0	0.5	1.3	14.0	0.6	5.0	1.5	18.4	0.7	5.2	1.1	11.3	0.8	6.6
Spain	16.5	5.9	21.1	7.9	58.0	28.1	0.5	0.2	20.5	9.5	9.5	3.4	44.3	21.4	11.5	3.9	18.1	8.2	14.7	5.6
**Western Europe**	**65.5**	**5.4**	**119.4**	**10.1**	**319.7**	**35.5**	**3.5**	**0.4**	**104.6**	**11.2**	**56.6**	**4.6**	**153.4**	**16.8**	**55.3**	**4.3**	**83.5**	**8.7**	**62.1**	**5.5**
Austria	2.5	4.8	5.0	9.9	13.8	38.2	0.1	0.3	4.3	11.2	2.5	4.7	5.7	15.7	2.2	4.1	3.4	8.1	2.7	5.6
Belgium	3.8	5.7	6.3	9.7	16.7	35.3	0.1	0.2	4.6	9.1	2.8	4.2	8.4	17.4	3.2	4.6	3.9	7.0	3.1	4.9
France	27.0	6.5	39.4	9.9	90.6	31.1	1.3	0.4	32.4	10.8	17.2	4.1	60.4	20.6	16.7	3.8	28.0	9.1	20.7	5.5
Germany	24.4	4.5	53.3	10.1	155.1	37.0	1.6	0.4	52.0	12.0	27.7	4.9	60.2	14.3	25.9	4.4	38.2	8.8	29.0	5.8
Luxembourg	0.2	5.4	0.4	13.5	0.6	31.4	0.0	0.0	0.1	5.7	0.1	3.0	0.4	17.4	0.1	4.3	0.2	6.8	0.1	4.8
Netherlands	5.4	5.7	10.7	11.4	29.8	42.9	0.2	0.3	8.4	10.7	4.7	5.0	13.0	17.9	5.1	5.3	6.3	7.5	4.1	4.5
Switzerland	2.3	4.7	4.5	9.6	13.0	36.7	0.1	0.3	2.7	7.1	1.7	3.5	5.2	14.4	2.1	4.2	3.5	8.7	2.3	5.0

Country/region	Thyroid				Hodgkin lymphoma				NHL				Multiple myeloma				Leukaemia				All sites			
	C73				C81				C82–86, C96				C88+C90				C91–95				C00–97/C44			
	M		F		M		F		M		F		M		F		M		F		M		F	
	Cases	ASR	Cases	ASR	Cases	ASR	Cases	ASR	Cases	ASR	Cases	ASR	Cases	ASR	Cases	ASR	Cases	ASR	Cases	ASR	Cases	ASR	Cases	ASR
**Europe**	**23.6**	**0.8**	**40.4**	**0.9**	**22.8**	**0.7**	**16.7**	**0.4**	**272.3**	**9.0**	**224.6**	**5.1**	**171.0**	**5.7**	**153.9**	**3.5**	**343.5**	**11.4**	**279.2**	**6.4**	**10765.9**	**347.8**	**8659.7**	**199.5**
**EU-27**	**15.6**	**0.8**	**24.3**	**0.9**	**12.7**	**0.6**	**9.0**	**0.3**	**193.0**	**9.5**	**155.9**	**5.6**	**124.8**	**6.2**	**108.0**	**3.9**	**241.9**	**12.0**	**192.4**	**6.9**	**7060.7**	**344.9**	**5556.5**	**204.5**
**Central and Eastern Europe**	**8.0**	**0.8**	**17.3**	**1.1**	**9.5**	**0.8**	**7.7**	**0.5**	**67.0**	**6.5**	**59.7**	**3.7**	**36.5**	**3.7**	**41.8**	**2.6**	**97.2**	**9.8**	**89.7**	**5.6**	**3796.7**	**378.2**	**3116.6**	**192.1**
Belarus	0.1	0.2	0.3	0.6	0.3	0.8	0.3	0.5	2.4	7.5	2.2	4.1	1.2	4.3	1.4	2.6	3.3	11.4	3.3	6.3	125.7	410.2	86.0	163.8
Bulgaria	0.2	0.5	0.3	0.6	0.3	1.1	0.2	0.5	2.0	6.2	1.4	3.3	0.8	2.3	0.7	1.5	2.7	8.8	2.2	5.2	110.9	350.0	82.3	194.1
Czechia	0.3	0.7	0.5	0.8	0.3	0.5	0.3	0.5	3.3	7.6	2.8	4.6	2.3	5.3	2.0	3.4	5.2	12.2	4.1	6.8	149.9	339.7	123.1	206.3
Hungary	0.4	0.9	0.6	1.0	0.2	0.5	0.2	0.3	3.1	7.8	3.3	5.6	1.4	3.6	1.5	2.4	4.5	11.7	4.1	6.9	172.7	437.3	153.7	261.6
Republic of Moldova	0.1	0.8	0.2	1.0	0.2	0.8	0.1	0.5	1.0	6.5	0.8	4.2	0.3	2.2	0.4	1.8	0.9	6.4	0.7	3.4	49.6	387.3	33.3	183.9
Poland	1.3	0.9	3.0	1.4	1.1	0.7	0.8	0.4	13.6	9.3	10.8	5.2	8.6	6.1	8.9	4.2	18.0	13.3	15.9	7.6	647.5	455.3	535.6	254.2
Romania	0.6	0.7	1.1	1.0	0.5	0.6	0.4	0.3	4.2	4.9	3.7	3.4	2.7	3.3	2.5	2.3	7.3	9.1	5.5	5.1	315.9	387.5	223.9	206.0
Russia	4.0	0.9	8.6	1.1	4.7	0.8	3.9	0.5	27.4	5.7	26.0	3.3	14.5	3.0	18.8	2.3	40.7	8.7	41.3	5.2	1662.3	361.7	1443.0	182.3
Slovakia	0.1	0.6	0.3	1.0	0.3	1.2	0.2	0.6	2.3	12.8	2.1	7.7	1.4	7.8	1.8	6.4	2.6	13.4	2.7	10.1	90.7	495.8	71.3	262.6
Ukraine	1.0	0.6	2.5	1.0	1.7	0.9	1.4	0.6	7.9	4.6	6.7	2.7	3.3	2.0	4.0	1.6	12.0	7.3	10.0	4.0	471.6	304.9	364.5	144.0
**Northern Europe**	**2.8**	**0.6**	**5.0**	**0.9**	**3.1**	**0.6**	**2.1**	**0.4**	**46.0**	**10.0**	**37.1**	**6.3**	**31.6**	**6.9**	**24.5**	**4.2**	**48.2**	**10.5**	**36.3**	**6.2**	**1456.9**	**317.5**	**1284.3**	**223.9**
Denmark	0.1	0.5	0.3	0.9	0.2	0.8	0.1	0.3	2.0	7.7	1.4	4.4	1.9	7.3	1.3	4.1	3.2	12.6	2.6	8.1	91.8	355.9	81.0	257.7
Estonia	0.0	0.8	0.1	1.5	0.0	0.7	0.0	0.2	0.6	12.3	0.5	5.3	0.4	7.9	0.4	4.4	0.7	15.9	0.6	7.2	21.4	456.8	18.4	209.5
Finland	0.3	1.1	0.3	0.9	0.2	0.8	0.2	0.4	2.7	10.5	2.2	6.1	1.8	6.9	1.6	4.6	2.2	8.6	1.7	5.0	71.5	274.7	61.9	180.3
Iceland	0.0	3.4	0.0	1.4	0.0	0.0	0.0	0.0	0.1	6.6	0.1	5.6	0.1	8.0	0.0	2.1	0.1	9.7	0.1	4.8	3.5	270.9	2.9	201.7
Ireland	0.1	0.8	0.3	1.4	0.2	1.0	0.1	0.6	1.9	11.7	1.6	8.3	1.3	8.5	0.9	4.8	1.7	10.5	1.3	6.6	54.3	343.2	48.7	253.5
Latvia	0.1	1.4	0.2	1.5	0.1	0.9	0.1	0.7	0.6	8.1	0.7	5.3	0.4	5.3	0.4	3.4	0.9	12.7	1.1	8.3	30.1	433.7	27.6	214.3
Lithuania	0.1	0.8	0.2	1.0	0.1	0.5	0.1	0.4	0.8	7.6	0.8	4.2	0.6	5.9	0.7	3.8	1.3	12.6	1.6	8.0	44.7	430.2	36.5	197.9
Norway	0.2	0.8	0.3	1.2	0.1	0.6	0.1	0.3	1.8	8.2	1.4	5.1	1.5	7.2	1.4	5.2	2.3	10.8	1.5	5.8	63.7	297.6	55.6	211.4
Sweden	0.3	0.7	0.5	0.8	0.2	0.4	0.1	0.2	4.4	9.5	3.3	5.7	3.3	7.1	2.2	3.8	4.6	9.8	3.7	6.4	127.5	272.0	116.3	206.5
United Kingdom	1.5	0.5	2.8	0.8	2.0	0.7	1.3	0.4	31.1	10.5	25.1	6.8	20.2	6.9	15.5	4.2	31.1	10.5	22.0	6.0	944.3	318.8	832.0	231.1
**Southern Europe**	**4.9**	**0.6**	**7.6**	**0.8**	**5.6**	**0.7**	**4.2**	**0.4**	**61.8**	**8.3**	**51.0**	**5.1**	**42.3**	**5.7**	**37.7**	**3.8**	**80.9**	**11.0**	**63.5**	**6.4**	**2422.2**	**325.6**	**1778.6**	**182.5**
Albania	0.1	0.8	0.1	0.7	0.1	0.7	0.1	0.6	0.2	1.8	0.1	0.7	0.2	1.6	0.1	0.6	0.9	7.0	0.6	5.0	26.3	243.7	13.0	108.5
Bosnia and Herzegovina	0.1	0.8	0.2	1.1	0.1	0.9	0.1	0.7	0.6	4.5	0.5	2.9	0.4	2.7	0.4	1.9	1.2	8.8	0.9	5.0	54.4	397.5	37.0	211.8
Croatia	0.2	0.8	0.3	1.0	0.1	0.5	0.1	0.3	1.6	8.7	1.7	6.3	1.1	6.1	1.0	3.9	2.5	14.8	2.1	8.2	81.3	457.5	60.9	236.7
Cyprus	0.0	0.7	0.1	1.0	0.0	0.6	0.0	0.0	0.5	12.6	0.3	6.7	0.3	8.0	0.3	6.1	0.8	19.7	0.5	10.5	14.4	361.7	9.9	200.0
Greece	0.3	0.6	0.5	0.7	0.6	1.1	0.5	0.7	3.5	6.4	2.6	3.7	3.0	5.4	2.9	4.1	7.5	13.7	5.6	7.9	196.9	361.3	133.2	191.8
Italy	2.1	0.7	3.2	0.7	2.4	0.8	1.8	0.5	29.0	9.3	22.8	5.4	19.6	6.3	18.1	4.2	35.1	11.3	28.2	6.7	957.8	308.2	776.9	186.3
North Macedonia	0.0	0.3	0.1	0.7	0.1	0.7	0.1	0.5	0.2	2.8	0.2	2.3	0.2	2.0	0.1	0.8	0.6	6.8	0.4	4.6	25.7	339.2	16.3	179.2
Malta	0.0	0.0	0.0	1.6	0.0	0.0	0.0	0.3	0.2	10.1	0.1	5.1	0.1	3.0	0.1	3.5	0.1	6.9	0.1	3.9	5.4	275.7	4.2	168.9
Montenegro	0.0	0.0	0.0	0.6	0.1	1.7	0.0	0.4	0.2	9.4	0.1	3.3	0.1	4.0	0.1	2.5	0.3	13.9	0.2	8.2	10.3	458.7	7.3	246.8
Portugal	0.4	0.8	0.5	0.7	0.3	0.6	0.2	0.3	5.2	10.9	4.4	6.4	3.8	8.0	2.6	3.8	5.4	11.3	4.3	6.3	180.7	374.2	118.8	173.9
Serbia	0.3	0.9	0.5	1.0	0.5	1.3	0.3	0.7	2.6	7.0	2.5	5.4	1.6	4.3	1.7	3.6	4.0	11.2	3.1	6.8	156.5	425.1	121.7	261.5
Slovenia	0.1	0.9	0.1	0.8	0.0	0.3	0.1	0.4	1.7	19.2	1.4	10.4	0.6	7.2	0.6	4.3	1.5	17.1	1.1	8.4	37.2	407.6	28.3	225.1
Spain	1.3	0.6	2.1	0.7	1.3	0.6	1.0	0.4	16.2	7.7	14.3	5.0	11.4	5.5	9.7	3.4	21.1	10.1	16.3	5.7	673.5	316.8	449.9	161.2
**Western Europe**	**8.0**	**0.9**	**10.6**	**0.9**	**4.7**	**0.5**	**2.8**	**0.2**	**97.5**	**10.5**	**76.8**	**6.1**	**60.7**	**6.6**	**50.0**	**4.0**	**117.1**	**12.7**	**89.7**	**7.1**	**3090.1**	**330.3**	**2480.1**	**205.7**
Austria	0.5	1.3	0.6	1.2	0.1	0.3	0.2	0.3	4.1	10.6	3.0	5.7	2.2	5.7	1.9	3.6	4.5	11.7	3.5	6.8	122.0	311.9	101.5	198.0
Belgium	0.5	1.0	0.6	0.9	0.3	0.6	0.2	0.2	5.3	10.7	3.9	5.8	3.2	6.5	2.5	3.7	6.5	13.2	5.6	8.2	165.9	327.9	133.6	203.7
France	2.0	0.7	2.6	0.6	1.7	0.6	0.8	0.2	32.9	11.1	25.7	6.0	19.8	6.7	17.5	4.1	39.3	13.3	29.7	6.9	1044.4	348.2	802.0	198.2
Germany	3.5	0.8	5.3	0.9	1.8	0.4	1.1	0.2	42.8	10.0	35.2	6.2	27.6	6.4	21.4	3.8	52.7	12.4	41.6	7.3	1378.7	318.7	1130.7	208.0
Luxembourg	0.0	0.5	0.0	0.3	0.0	0.0	0.0	0.0	0.1	6.0	0.2	6.0	0.1	6.6	0.1	4.0	0.3	12.7	0.2	5.6	6.6	298.3	5.0	178.4
Netherlands	0.8	1.1	0.9	1.0	0.4	0.6	0.4	0.4	8.7	11.5	5.9	6.2	5.1	6.8	4.4	4.6	9.8	13.2	6.5	6.9	267.3	350.9	221.4	235.7
Switzerland	0.6	1.4	0.5	1.0	0.2	0.6	0.1	0.2	3.6	9.6	2.8	5.7	2.6	6.8	2.2	4.5	4.0	10.6	2.6	5.3	103.9	273.3	85.1	178.0

European areas are reported in bold.

a ASR = Age-standardised rates using European standard population 2013, based on estimated number of deaths; M = Males, F = Females; CNS = central nervous system; NHL = Non-Hodgkin lymphoma.

**Appendix Table A3:** Estimated numbers of new cancer cases (thousands), ASRs^a^ (per 100,000) by sex, cancer site, and age group in Europe for 2020.

Cancer	Both sexes									Males									Females								
	0–44			45–64			65+			0–44			45–64			65+			0–44			45–64			65+		
	Cases	%	ASR	Cases	%	ASR	Cases	%	ASR	Cases	%	ASR	Cases	%	ASR	Cases	%	ASR	Cases	%	ASR	Cases	%	ASR	Cases	%	ASR
Lip, oral cavity and pharynx	75.0	2.7	1.8	619.2	4.6	29.7	592.1	2.5	41.4	54.0	5.5	2.7	483.7	6.9	48.4	395.6	3.0	66.2	21.0	1.2	1.0	135.5	2.0	12.5	196.5	1.8	23.1
Oesophagus	9.9	0.4	0.2	196.2	1.4	9.3	323.8	1.3	22.7	7.6	0.8	0.4	162.1	2.3	16.2	234.4	1.8	40.2	2.3	0.1	0.1	34.1	0.5	3.1	89.3	0.8	10.4
Stomach	39.8	1.4	1.0	397.5	2.9	18.9	923.0	3.8	64.5	19.9	2.0	1.0	272.0	3.9	27.1	545.3	4.1	94.6	19.9	1.1	1.0	125.5	1.9	11.5	377.8	3.5	43.8
Colorectum	118.2	4.2	2.9	1482.8	10.9	70.6	3597.2	15.0	252.1	52.3	5.4	2.6	852.8	12.3	85.1	1912.1	14.3	333.6	66.0	3.7	3.3	630.0	9.5	57.5	1685.1	15.8	196.8
Liver	20.2	0.7	0.5	260.9	1.9	12.4	595.1	2.5	41.8	13.3	1.4	0.6	200.5	2.9	20.0	367.0	2.7	63.5	7.0	0.4	0.3	60.4	0.9	5.5	228.2	2.1	26.5
Gallbladder	1.4	0.1	0.0	25.8	0.2	1.2	98.5	0.4	6.9	0.6	0.1	0.0	8.6	0.1	0.9	29.6	0.2	5.3	0.9	0.0	0.0	17.2	0.3	1.6	68.8	0.6	8.0
Pancreas	19.4	0.7	0.5	360.1	2.7	17.1	1021.7	4.2	71.5	11.3	1.2	0.6	218.6	3.1	21.8	472.2	3.5	82.6	8.1	0.4	0.4	141.5	2.1	12.8	549.5	5.1	63.6
Larynx	10.0	0.4	0.2	193.7	1.4	9.2	194.9	0.8	13.6	8.2	0.8	0.4	171.0	2.5	17.1	170.3	1.3	28.5	1.8	0.1	0.1	22.7	0.3	2.1	24.6	0.2	2.9
Lung	57.0	2.0	1.4	1629.8	12.0	77.3	3088.5	12.8	216.9	30.7	3.1	1.5	1093.1	15.7	108.9	2026.7	15.2	348.2	26.3	1.5	1.3	536.7	8.1	48.8	1061.8	9.9	126.6
Melanoma of skin	244.0	8.8	6.0	543.3	4.0	26.2	719.0	3.0	50.4	93.4	9.6	4.6	272.4	3.9	27.3	397.3	3.0	69.1	150.6	8.3	7.5	270.9	4.1	25.3	321.7	3.0	38.0
Breast																			628.1	34.8	30.7	2274.0	34.3	211.6	2408.8	22.5	287.0
Cervix uteri																			198.4	11.0	9.8	245.0	3.7	23.2	138.3	1.3	16.5
Corpus uteri																			47.3	2.6	2.3	569.8	8.6	52.2	683.4	6.4	82.3
Ovary																			71.9	4.0	3.5	259.0	3.9	24.0	336.0	3.1	39.8
Prostate										5.0	0.5	0.2	1349.8	19.4	134.0	3378.6	25.3	576.8									
Testis										180.3	18.5	8.8	58.8	0.8	6.0	11.5	0.1	1.9									
Kidney	79.0	2.8	1.9	534.4	3.9	25.6	772.7	3.2	54.2	50.6	5.2	2.5	356.3	5.1	35.7	451.4	3.4	77.5	28.5	1.6	1.4	178.1	2.7	16.3	321.3	3.0	37.9
Bladder	25.1	0.9	0.6	516.5	3.8	24.5	1498.2	6.2	105.1	15.9	1.6	0.8	402.1	5.8	40.1	1148.6	8.6	202.5	9.2	0.5	0.5	114.4	1.7	10.4	349.6	3.3	40.6
Brain, central nervous system	126.2	4.5	3.1	242.2	1.8	11.7	302.7	1.3	21.2	73.8	7.6	3.6	138.1	2.0	13.9	150.0	1.1	25.7	52.5	2.9	2.6	104.1	1.6	9.6	152.7	1.4	18.0
Thyroid	270.3	9.7	6.7	381.5	2.8	18.5	219.8	0.9	15.4	50.4	5.2	2.5	88.2	1.3	8.9	54.9	0.4	9.2	219.9	12.2	11.0	293.3	4.4	27.6	165.0	1.5	20.1
Hodgkin lymphoma	105.9	3.8	2.7	49.3	0.4	2.4	43.3	0.2	3.0	56.6	5.8	2.8	29.8	0.4	3.0	23.6	0.2	4.1	49.3	2.7	2.5	19.5	0.3	1.8	19.8	0.2	2.3
Non-Hodgkin lymphoma	119.8	4.3	3.0	386.0	2.8	18.5	724.0	3.0	50.8	72.7	7.4	3.6	225.4	3.2	22.6	375.7	2.8	65.7	47.1	2.6	2.3	160.6	2.4	14.8	348.3	3.3	40.9
Multiple myeloma	10.2	0.4	0.3	142.4	1.0	6.8	356.5	1.5	25.0	6.2	0.6	0.3	81.7	1.2	8.2	190.4	1.4	33.5	4.0	0.2	0.2	60.7	0.9	5.5	166.1	1.6	19.6
Leukaemia	132.4	4.8	3.3	259.9	1.9	12.4	607.9	2.5	42.6	73.2	7.5	3.6	153.4	2.2	15.3	331.4	2.5	58.5	59.3	3.3	3.0	106.5	1.6	9.8	276.5	2.6	32.1
All sites excl. non-melanoma skin	2784.0		68.5	13585.3		649.2	24053.3		1687.0	976.7		47.8	6961.1		694.6	13364.1		2309.9	1807.3		89.3	6624.3		611.7	10689.3		1260.3

a ASR = Age-standardised rates using European standard population 2013, based on estimated number of deaths.

**Appendix Table A4:** Estimated numbers of deaths from cancer (thousands), ASRs^a^ (per 100,000) by sex, cancer site, and age group in Europe for 2020.

Cancer	Both									Males									Females								
	0–44			45–64			65+			0–44			45–64			65+			0–44			45–64			65+		
	Deaths	%	ASR	Deaths	%	ASR	Deaths	%	ASR	Deaths	%	ASR	Deaths	%	ASR	Deaths	%	ASR	Deaths	%	ASR	Deaths	%	ASR	Deaths	%	ASR
Lip, oral cavity and pharynx	18.6	3.8	0.5	241.6	5.0	11.6	278.6	2.0	19.4	14.8	6.8	0.7	204.0	7.2	20.4	195.4	2.5	33.1	3.8	1.4	0.2	37.6	1.9	3.4	83.2	1.3	9.6
Oesophagus	5.6	1.2	0.1	151.7	3.2	7.2	297.8	2.1	20.9	4.4	2.0	0.2	128.9	4.6	12.9	215.8	2.8	37.4	1.1	0.4	0.1	22.8	1.2	2.1	82.0	1.3	9.5
Stomach	24.9	5.1	0.6	248.3	5.2	11.8	696.8	4.9	48.6	12.4	5.7	0.6	172.9	6.1	17.2	409.3	5.3	71.7	12.5	4.6	0.6	75.4	3.8	6.9	287.5	4.5	33.0
Colorectum	29.4	6.0	0.7	463.3	9.7	22.0	1955.5	13.8	136.6	14.0	6.4	0.7	277.9	9.8	27.7	1027.0	13.3	183.5	15.5	5.7	0.8	185.5	9.4	16.9	928.5	14.5	106.0
Liver	10.9	2.2	0.3	201.0	4.2	9.6	572.2	4.0	40.1	7.2	3.3	0.4	151.4	5.4	15.1	354.9	4.6	62.3	3.7	1.4	0.2	49.7	2.5	4.5	217.3	3.4	25.0
Gallbladder	0.5	0.1	0.0	16.3	0.3	0.8	70.3	0.5	4.9	0.2	0.1	0.0	5.3	0.2	0.5	20.5	0.3	3.7	0.3	0.1	0.0	11.1	0.6	1.0	49.8	0.8	5.8
Pancreas	13.3	2.7	0.3	322.0	6.7	15.3	986.0	7.0	69.0	8.6	4.0	0.4	199.8	7.1	19.9	458.5	5.9	80.4	4.7	1.7	0.2	122.2	6.2	11.0	527.5	8.2	61.0
Larynx	3	0.6	0.1	79.3	1.7	3.8	113.7	0.8	7.9	2.6	1.2	0.1	72.2	2.6	7.2	101.3	1.3	17.3	0.5	0.2	0.0	7.1	0.4	0.6	12.3	0.2	1.4
Lung	31.3	6.4	0.8	1176.4	24.5	55.7	2634.1	18.6	184.7	18.3	8.4	0.9	823.1	29.2	81.9	1758.9	22.8	305.0	13.0	4.8	0.6	353.3	17.9	32.0	875.3	13.7	103.5
Melanoma of skin	18.5	3.8	0.5	70.7	1.5	3.4	174.4	1.2	12.2	10.0	4.6	0.5	41.4	1.5	4.1	95.7	1.2	17.0	8.5	3.2	0.4	29.3	1.5	2.7	78.7	1.2	9.0
Breast																			67.6	25.1	3.3	381.8	19.3	35.2	968.3	15.1	111.6
Cervix uteri																			41.0	15.2	2.0	107.3	5.4	10.0	111.6	1.7	13.1
Corpus uteri																			3.3	1.2	0.2	69.6	3.5	6.3	226.8	3.5	26.5
Ovary																			15.3	5.7	0.8	136.5	6.9	12.5	288.6	4.5	33.9
Prostate										0.7	0.3	0.0	89.4	3.2	8.9	990.8	12.8	184.0									
Testis										8.3	3.8	0.4	3.8	0.1	0.4	3.5	0.0	0.6									
Kidney	8.2	1.7	0.2	130.6	2.7	6.2	401.8	2.8	28.1	5.5	2.5	0.3	96.4	3.4	9.6	244.1	3.2	43.2	2.7	1.0	0.1	34.2	1.7	3.1	157.7	2.5	18.1
Bladder	2.2	0.5	0.1	80.3	1.7	3.8	590.4	4.2	41.2	1.2	0.6	0.1	63.4	2.2	6.3	443.5	5.7	81.1	1.0	0.4	0.0	16.9	0.9	1.5	146.8	2.3	16.5
Brain, central nervous system	59.4	12.2	1.5	196.3	4.1	9.4	281.1	2.0	19.7	36.4	16.8	1.8	116.0	4.1	11.6	141.3	1.8	24.3	23.0	8.5	1.1	80.3	4.1	7.4	139.8	2.2	16.5
Thyroid	1.2	0.2	0.0	13.5	0.3	0.6	49.3	0.3	3.4	0.6	0.3	0.0	6.7	0.2	0.7	16.4	0.2	2.9	0.6	0.2	0.0	6.8	0.3	0.6	33.0	0.5	3.8
Hodgkin lymphoma	8.3	1.7	0.2	10.2	0.2	0.5	21.0	0.1	1.5	5.1	2.3	0.2	6.7	0.2	0.7	11.0	0.1	1.9	3.2	1.2	0.2	3.6	0.2	0.3	10.0	0.2	1.2
Non-Hodgkin lymphoma	18.9	3.9	0.5	87.8	1.8	4.2	390.2	2.8	27.3	11.9	5.5	0.6	55.7	2.0	5.6	204.7	2.6	36.8	7.0	2.6	0.3	32.0	1.6	2.9	185.6	2.9	21.2
Multiple myeloma	1.8	0.4	0.0	56.1	1.2	2.7	267.1	1.9	18.7	1.1	0.5	0.1	31.5	1.1	3.1	138.4	1.8	24.9	0.7	0.3	0.0	24.6	1.2	2.2	128.7	2.0	14.9
Leukaemia	35	7.2	0.9	102.3	2.1	4.9	485.3	3.4	33.9	20.0	9.2	1.0	59.7	2.1	6.0	263.8	3.4	47.5	15.1	5.6	0.8	42.6	2.2	3.9	221.5	3.5	25.3
All sites excl. non-melanoma skin	486.9		12.0	4800.2		228.3	14138.4		988.3	217.3		10.6	2822.2		281.4	7726.3		1371.5	269.6		13.3	1977.9		180.6	6412.2		740.8

a ASR = Age-standardised rates using European standard population 2013, based on estimated number of new cases.
